# Human cerebral organoids model tumor initiation and infiltration in an autologous astrocyte-supported setting

**DOI:** 10.1016/j.isci.2025.113334

**Published:** 2025-08-11

**Authors:** Esther Schickel, Tamara Bender, Leon Kaysan, Simone Hufgard, Margot Mayer, David R. Grosshans, Christiane Thielemann, Insa S. Schroeder

**Affiliations:** 1 Biophysics Department, GSI Helmholtzzentrum für Schwerionenforschung GmbH, Darmstadt, Hessen, Germany; 2 BioMEMS Lab, University of Applied Sciences Aschaffenburg, Aschaffenburg, Bayern, Germany; 3 Department of Radiation Oncology, Division of Radiation Oncology, The University of Texas MD Anderson Cancer Center, Houston, TX, USA

**Keywords:** Developmental neuroscience, Cancer, Experimental models in systems biology

## Abstract

Efforts to efficiently target brain tumors are constrained by the dearth of appropriate models to study tumor behavior toward treatment approaches as well as potential side effects to the surrounding normal tissue. We established a reproducible cerebral organoid model of brain tumorigenesis in an autologous setting by overexpressing *c-MYC*, a common oncogene in brain tumors. GFP^+^/c-MYC^high^ cells were isolated from tumor organoids and used in two different approaches: GFP^+^/c-MYC^high^ cells co-cultured with cerebral organoid slices or fused as spheres to whole organoids. GFP^+^/c-MYC^high^ cells used in both approaches exhibited tumor-like properties, including an immature phenotype and a highly proliferative and invasive potential. We demonstrate that the latter is influenced by astrocytes supporting the GFP^+^/c-MYC^high^ cells while X-ray irradiation significantly kills and impairs tissue infiltration of GFP^+^/c-MYC^high^ cells. In summary, the model represents major features of tumorous and adjacent normal tissue and may be used to evaluate appropriate cancer treatments.

## Highlights

• C-MYC^high^ human organoids model brain tumorigenesis in an autologous setting
• Assembloids (c-MYC^high^ tumor spheres fused with organoids) resemble tumor margin
• Organoid slices co-cultured with c-MYC^high^ cells enable long-term therapy studies
• Astrocytes support c-MYC^high^ cells, X-rays diminish their number and invasion

## Graphical abstract

**Figure:**
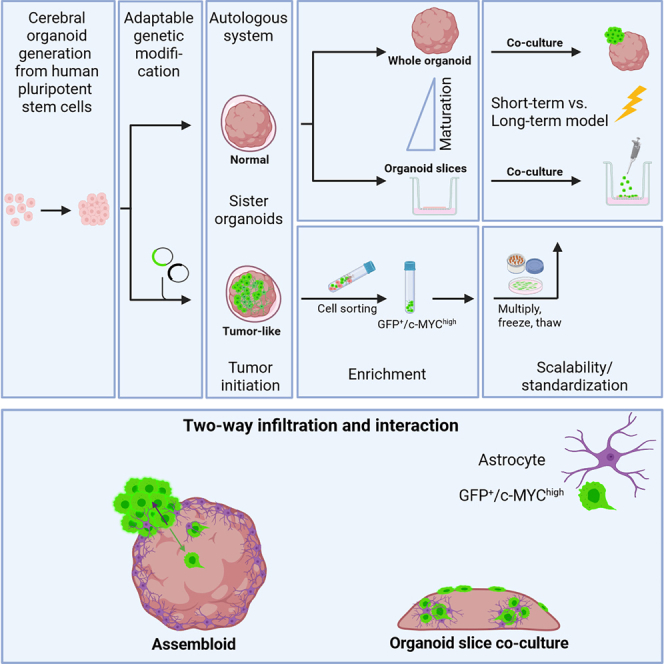

## Introduction

Malignant brain tumors present a significant societal burden, as effective therapeutic options remain limited for many tumor types, despite extensive research efforts in surgery, chemotherapy, immunotherapy, and radiotherapy, with treatment outcomes depending on multiple parameters such as the tumor type, size, grade, and location. For instance, the prognosis for glioblastoma patients remains poor, with a median survival of approximately 15 months.^1^^,^^2^ Pediatric patients are particularly affected, as they do not respond well to most of the standard treatments.^3^^,^^4^^,^^5^ The reconsideration of treatment modalities necessitates the development of human tissue and organ models more appropriate than those previously described.^6^ For example, a monolayer of tumor cells in a dish or *trans*-well insert can be employed for studies of tumor migration,^7^^,^^8^ but not infiltration due to the lack of a three-dimensional environment. Furthermore, the loss of cellular diversity resulting from clonal selection during cell propagation and increased genetic variation within cell lines represent additional drawbacks of such cultures.^9^ This, in turn, diminishes their physiological relevance. Animal models, such as rats and mice, can be used to inject tumor cells, to be xenografted with a patient’s tumor tissue, or allografted with genetically modified cells.^10^^,^^11^ However, these models fail to accurately recapitulate the situation in humans due to species differences in the genetic background, morphology, anatomy, and metabolic states.^12^ Regarding the potential use of human cells, one or more cell types can be aggregated and maintained as uniform spheres. However, they lack the complexity, which is observed in organoids. Conversely, cerebral organoids derived from pluripotent stem cells comprise of diverse cell types, including neuronal and glial cells, and represent a promising platform for mimicking human brain tumors *in vitro*. A variety of approaches have been employed to achieve this goal, including the use of patient-derived explants of brain tumors cultured in scaffolds or matrices or dissociated tumor cells that can self-arrange into patient-derived organoids (PDOs),^13^^,^^14^^,^^15^^,^^16^ and be used to study tumor invasion in personalized drug screenings. Unfortunately, such models are not suitable for long-term culture due to the loss of tumor heterogeneity during their propagation.^17^^,^^18^ To overcome this, patient-derived tumor explants of brain tumors of different origins and malignancy grades can be cultured within iPSC-derived cerebral organoids enabling preservation of patient tumor molecular pathology and cellular diversity.^19^ Further approaches described in the literature include the co-culture of cerebral organoids with primary tumor cells or cell lines.^20^ However, the genetic and epigenetic landscapes of each entity differ, which presents a challenge for the formation of the tumor in its original surrounding. Genetic manipulations of oncogenes and tumor suppressor genes can be induced in the organoid itself by tools such as CRISPR-Cas9 and/or the *Sleeping Beauty* transposon system to enable the formation of the tumor in its original surrounding.^21^^,^^22^ Here, we present an adaptation and combination of existing protocols, resulting in an autologous brain tumor model in which long-term culture, as well as the study of effects at the margin between tumor and normal tissue, is possible. For this purpose, normal cerebral organoids were generated using human pluripotent stem cells (hPSCs) and a protocol for their 3D organization and differentiation into neuronal and glial cells.^23^^,^^24^ In addition, tumor organoids containing genetically modified cells were generated using the *Sleeping Beauty* transposon system for the induction of *c-MYC* oncogene overexpression.^22^ During prolonged culture periods, organoids typically develop a necrotic core due to inadequate supply of oxygen and nutrients from the medium.^25^ This renders organoids unsuitable for studies aimed at observing long-term therapy effects. To circumvent this issue, we prepared 300 μm organoid slices and cultured them at air-liquid interface (ALI), which provided a dual supply of nutrients from the medium and oxygen from the air. This enabled us to achieve long-term cultures^26^ beyond 100 days without the occurrence of necrosis. To generate a reproducible tumor model in an autologous setting, we either co-cultured organoid slices with a defined number of tumor-like cells isolated from sister organoids using fluorescence-activated cell sorting (FACS) based on the presence of a GFP signal or used them to generate uniform tumor-spheres that were fused with whole sister organoids to form assembloids. As radiotherapy is one component of the standard of care for brain tumors in adults^27^ and often unavoidable in pediatric patients,^28^^,^^29^ we tested the tumor model using X-ray irradiation. Tumor-like cells interacted with normal cells from the organoids or organoid slices, e.g., astrocytes that supported their proliferation, migration, and infiltration. Upon irradiation, significant cell killing and impaired tissue infiltration of tumor-like cells was observed.

## Results

**Figure 1:**
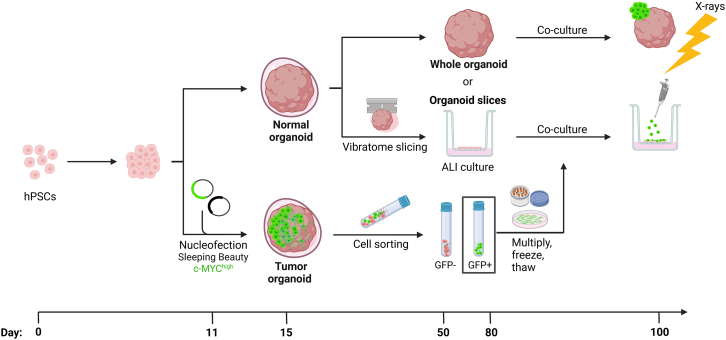
Workflow for generation of the tumor model: tumor spheres assembled with cerebral organoids and cerebral organoid slices co-cultured with tumor-like cells at air liquid interface Generation of embryoid bodies using human pluripotent stem cells (hPSCs). Nucleofection of organoids on day 11 of the culture using *Sleeping Beauty* transposon system for overexpression of *c-MYC* oncogene with co-expressed *GFP*. Embedding of organoids in Matrigel on day 15 of the culture. Normal organoids cultured as whole entities or 300 μm thick organoid slices prepared using vibratome at ∼ day 50 and maintained on cell culture inserts. Normal organoids or organoid slices cultured in parallel with nucleofected organoids used for isolation of genetically modified cells based on the presence of GFP signal using fluorescence-activated cell sorting at ∼ day 50 for co-culture with organoid slices or at ∼ day 80 for generation of tumor spheres to be assembled with whole organoids. Positively selected (GFP^+^) cells can be propagated, frozen, and thawed before used for either generation of spheres to be assembled with whole organoid or as single cell suspension applied directly on top of the slice at ALI-culture. Created with BioRender.com.

We established an autologous brain tumor model by generating cerebral organoids combining previously published protocols.^23^^,^^24^ The workflow is shown schematically in Figure 1: Cerebral organoids derived from hPSCs were cultured either as intact structures (whole organoids) or as 300 μm thick, vibratome-cut organoid slices to allow long-term culture via improved nutritional support in ALI conditions.^26^ In parallel, tumor organoids were generated by randomly introducing GFP-tagged *c-MYC* overexpression via nucleofection using the *Sleeping Beauty* transposon system. GFP^+^/c-MYC^high^ cells were isolated from the resulting tumor organoids through cell sorting, then either directly used, expanded in culture or cryopreserved for later use. This approach permits the controlled and reproducible generation of an autologous tumor model by either generating assembloids from whole normal organoids fused with tumor spheres—formed from a defined number of GFP^+^/c-MYC^high^ tumor-like cells—or by co-culturing organoid slices with a distinct number of tumor-like cells (Figure 1). The characteristics of whole organoids and organoid slices were evaluated, including cell composition and interactions as well as the dynamics that enable cellular processes and functions in these structures. Tumor-like properties of transfected, GFP^+^/c-MYC^high^ cells were confirmed, including oncogene overexpression, immature or stem cell-like properties, highly proliferative and invasive potential.

### Cerebral organoids exhibit dynamic transition from proliferation to maturation

**Figure 2:**
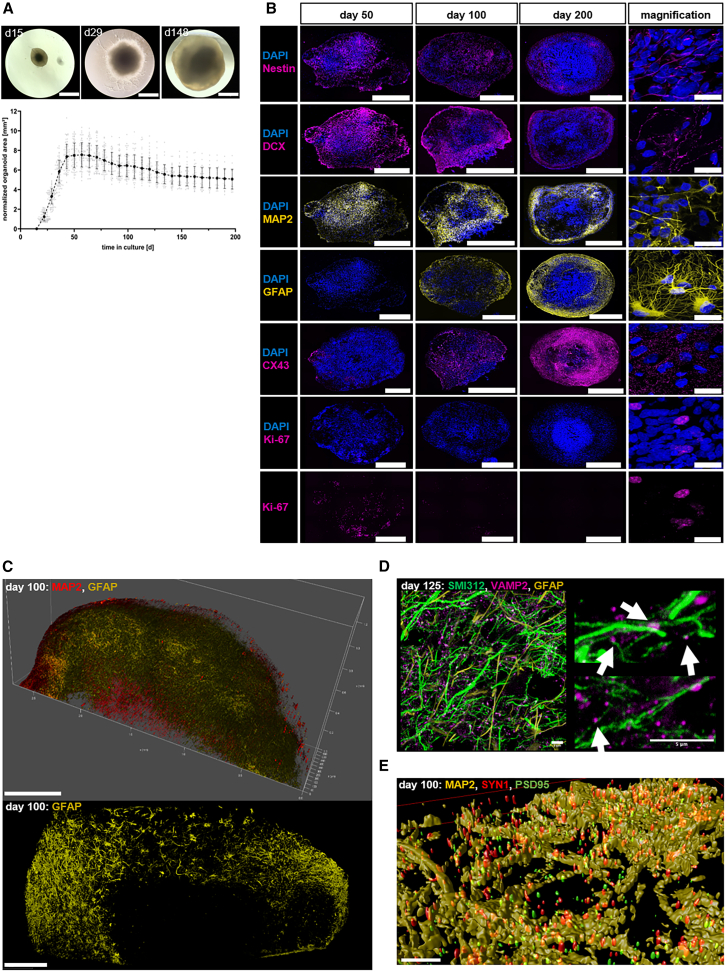
Proliferation and maturation in cerebral organoids (A) Representative microscopy images of organoids at day 15, 29, and 148 of the culture. The organoid size measured as circular area (mm^2^) for organoids between day 15 and 200 of the culture, normalized to day 15. Data are presented as mean ± SD for three independent experiments (*N* = 3) and 24 to 104 organoids per experiment (*n* = 24–104). Scale bars: 1000 μm. (B) Representative immunofluorescence staining of nestin (magenta), DCX (magenta), MAP2 (yellow), GFAP (yellow), CX43 (magenta), and Ki-67 (magenta) for organoids at day 50, 100, and 200 of the culture, respectively. Scale bars: 1000 μm. Magnification for nestin at day 50, DCX at day 100, MAP2 at day 100, GFAP at day 150, CX43 at day 200, and Ki-67 at day 50 of the culture. Scale bars: 20 μm. (C) Representative immunofluorescence staining of SYN1 (red) in combination with MAP2 (yellow) (700 μm z stack), and of GFAP (yellow) (overview images) at day 100 of the culture. Scale bars: 500 μm for SYN1 and MAP2, 200 μm for GFAP staining. (D) Magnification image of representative immunofluorescence staining of SMI312 (green), VAMP2 (magenta; indicated with white arrows (right image) and GFAP (yellow) at day 125 of the culture. Scale bars: 5 μm. (E) Magnification image of representative immunofluorescence staining of HOMER1 (green), VAMP2 (red), MAP2 (yellow) at day 125 of the culture after deconvolution (right), and surface rendering (left). Scale bars: 3 μm. See also Figures S1 and S2.

Cerebral organoids generated from human embryonic stem cells showed an increase in size during the culture period as evidenced by the measurement of the organoids circular area. This was attributed to intense cell proliferation as demonstrated by the increase in the measured organoid circular area and by the detection of Ki-67 positive cells up to approximately day 50 in culture, after which no further increase in organoid size and little to no Ki-67 signal was observed (Figures 2A and 2B). Notably, different cell types were present in the organoids at the same time, but the protein expression of their specific markers and their spatial distribution was dependent on the organoids age (Figure 2B shows spatial distribution while Figure S1 displays quantification of the IF signal of selected markers). For instance, early progenitors expressing nestin were present at approximately day 25 with distribution throughout the organoid. Beyond day 100, nestin-expressing cells were predominantly found at the outer edge of the organoid. Neuronal markers such as MAP2 and DCX were also detectable before day 50 of differentiation and were expressed throughout the organoid. Their expression peaked around day 50, after which it slightly decreased and the MAP2 and DCX positive cells were predominantly found at the outer edge of the organoid, similar to the spatial distribution of nestin. SMI312 marking axons showed an increase in fluorescence intensity around day 50 of the culture, which continued beyond day 100 with no further significant increase in expression intensity. An increase in the abundance of astrocytes, as indicated by the expression of GFAP, started around day 100 and continued until day 150 at the outer edge of the organoid. The gap junction marker CX43, highly expressed in astrocytes, showed a similar expression pattern. However, here the increase in expression started slightly earlier at about day 75 but also reached its plateau at day 150 of the culture (Figures 2B and S1). In addition, the pre-synaptic markers VAMP2 and SYN1 were localized near neuronal cells (SMI312^+^ or MAP2^+^) at the outer edge of organoids where astrocytes (GFAP^+^) were also found at ∼ day 100–125 of the culture (Figures 2C–2E). The presynaptic marker SYN1, primarily localized to the synaptic vesicles in the axonal terminal and involved in neurotransmitter release, was observed in close proximity to the postsynaptic marker PSD95 (Figures 2E and S2).

### Cerebral organoids and organoid slices are robust models that exhibit brain-like features

**Figure 3:**
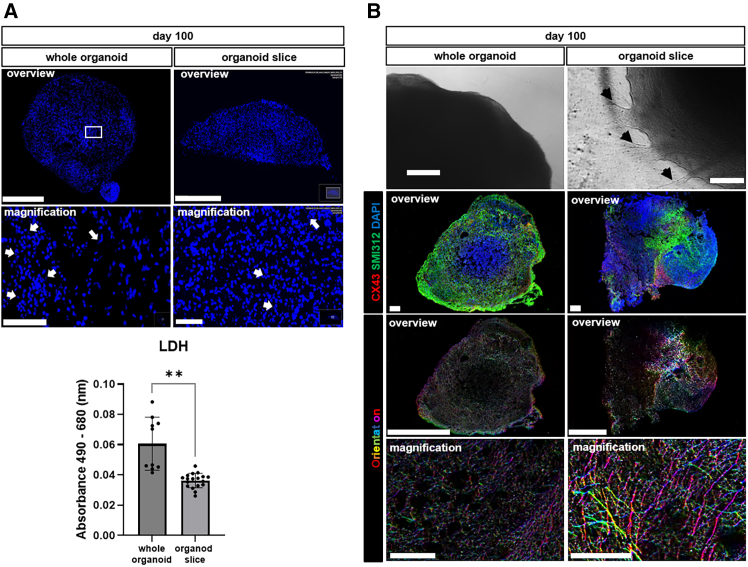
Whole organoids versus organoid slices cultured at the air-liquid interface (ALI) at day 100 of the culture (A) Representative DAPI staining of whole organoids cultured in medium and in organoid slices cut into 300 μm thick sections cultured on cell culture inserts at the ALI in overview and magnification. Scale bars: 1000 μm in overview and 100 μm in magnification (top). Lactate dehydrogenase (LDH) level measured in the medium of whole organoids or 300 μm thick organoid slices in ALI culture. Data are presented as mean ± SD for three to six independent experiments (N = 3–6) and 3 to 15 organoids per experiment (n = 3–15), ∗*p* < 0.05, ∗∗*p* < 0.01, ∗∗∗*p* < 0.001, ∗∗∗∗*p* < 0.0001. Statistical analysis was done using unpaired t test (bottom). (B) Representative phase contrast image of the whole organoid with smooth edges, and organoid slice showing protrusions extending from the main body of the organoid slice (black arrow). Scale bars: 320 μm. Representative immunofluorescence staining of CX43 (red) in combination with SMI312 (green) and color-coded visualized orientation for SMI312 staining (pseudocolor). Scale bars: 1000 μm in overview and 100 μm in magnification. See also Figures S3–S5.

An increase in organoid size during the culture period was correlated with the occurrence of cell death in the organoid interior, indicated by shrunken and fragmented nuclei in DAPI staining and measured as lactate dehydrogenase (LDH) release (Figure 3A). This was most likely due to an inadequate supply of oxygen and nutrients, which is a limiting factor for prolonged culture. In contrast to whole organoids, organoids cut into 300 μm thick slices and cultured in ALI conditions showed mostly intact nuclei and significantly less extracellular LDH than whole organoids of the same age while maintaining the original 3D architecture. The abundance of proliferative cells (Ki-67^+^) was low in whole organoids at day 100 of the culture and comparable to that in organoid slices of the same age (Figures S3A–S3C). The occurrence of apoptosis, as evidenced by caspase-3 active staining at day 100 was very low in both culture systems (Figures S3A–S3C).

**Figure 4:**
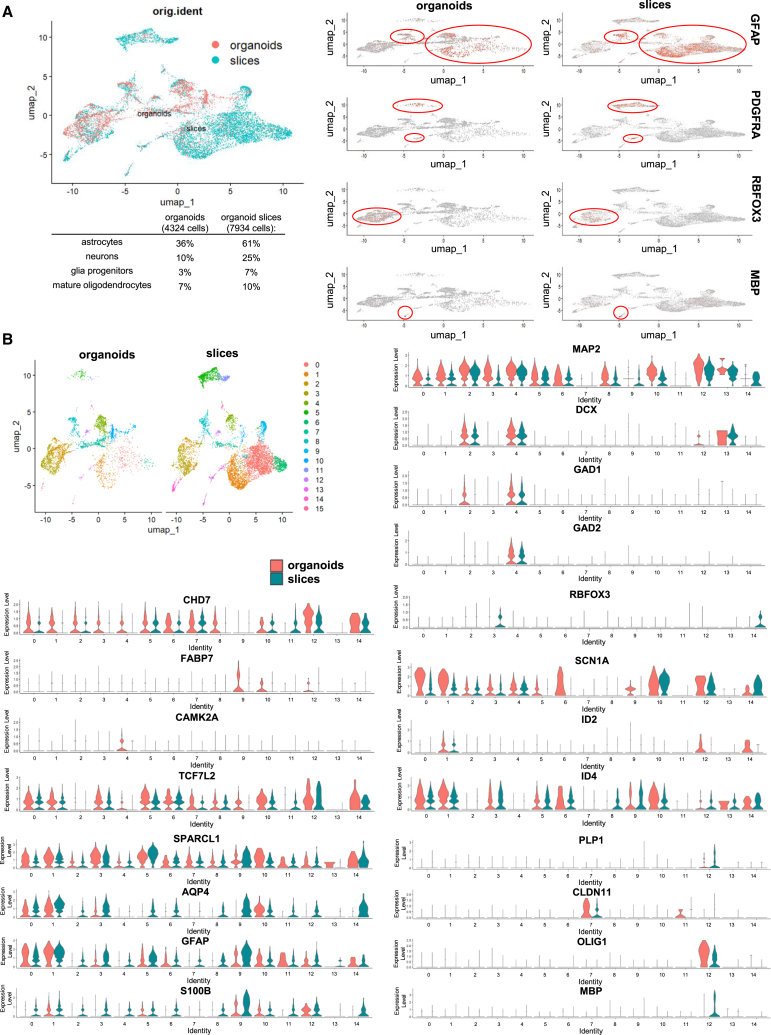
Whole organoids versus organoid slices at day 100 of the culture (A) Single nuclei (sn) RNA sequencing analysis of integrated datasets for whole organoids and organoid slices showing the distribution of key markers of glial and neuronal cells representing populations of astrocytes (*GFAP*), glial progenitors (*PDGFRA*), neurons (*RBFOX3*), and oligodendrocytes (*MBP*) encircled in red. (B) Comparison of markers of differentiation and maturation (*CHD7*, *FABP7*, *CAMK2A*, *TCF7L2*, *SPARCL1*, *AQP1*, *GFAP*, *S100B*, *MAP2*, *DCX*, *GAD1*, *GAD2*, *RBFOX3*, *SNC1A*, *ID2*, *ID4*, *PLP1*, *CLDN11*, *OLIG1*, and *MBP*) among Seurat clusters in organoids and organoid slices. See also Figure S5.

Organoid slices cultured at the ALI showed axon tracts extending away from the main body of the slice that grew in width and size over time (Figure 3B). Organoid slices were maintained for 100 days and beyond (Figure S5) and continued to display brain-like features, including dynamic and organized axonal networks, as evidenced by staining with SMI312 that revealed orientation of axon fibers in many different directions, in contrast to the little directionality observed in the color-coded visualized orientation of age-matched whole organoids (Figure 3B). Furthermore, intercellular networks were confirmed by staining for the gap junction protein CX43. At day 100, organoids and organoid slices exhibited a heterogeneous cellular composition (Figure S4), including glial precursor cells (GLAST^+^ and PDGFRα^+^), neurons (MAP2^+^), and radial glia and mature astrocytes (GFAP^+^). While there was no significant difference in the expression of *GFAP* between the two culture systems at the mRNA level, its spatial distribution in whole organoids predominantly at the outer area was different from organoid slices, in which it was more equally distributed (Figure S4A). Furthermore, organoid slices showed a higher expression of *MAP2* and lower expression of *VIM* (vimentin, an intermediate filament protein expressed by immature astrocytes^30^), compared to whole organoids at the mRNA level (Figure S4A). Notably, IF and single nuclei RNA sequencing analyses revealed that expression of PDGFRα/*PDGFRA*, a marker of oligodendrocytes^31^ and glia cell markers in general, were more prominent in organoid slices than in whole organoids, suggesting that ALI culture leads to enhanced cell maturation (Figures 4A and S4A). Transition and immature as well as more mature neuron markers (*DCX* (active migration state), *GAD1/GAD2* (subcortical/interneuron populations), *MAP2* (neural maturation), *RBFOX3* (mature neurons), and *SCN1A* (functional excitability, maturing neurons), and functional markers (*CAMK2A*, *SPARCL1*, and *TCFL2*), *ID2*, *ID4* (glial development and astrocyte specification)*,* and glial markers (*GFAP*, *S100B* (in astrocytes), *PLP1*, *OLIG1*, *CLDN11* (in early oligodendrocyte lineage/oligodendrocytes*)*, *MBP* (mature oligodendrocytes)), were expressed in organoids and organoids slices to different extents (Figure 4). While the core progenitor marker *CHD7* was present across nearly all clusters in both models, organoid slices showed enhanced neuronal and oligodendrocyte maturation (*RBFOX3*, *MBP*) (Figure 4). On the other hand, whole organoids retained more radial glia and early progenitor markers (*FABP7*, *ID2/ID4*). *CAMK2A* and *RBFOX3* suggest cortical neuron identity and define excitatory neuron maturation, with *CAMK2A* predominantly expressed in organoids, and *RBFOX3* in organoid slices (Figure 4). Such organization and composition enabled processes and functions such as myelination, which is mediated by MBP and spontaneous cell activity. Although the expression of *MBP* was significantly higher at the mRNA level in organoid slices compared to organoids at day 100, its protein expression was rather low in both model systems, as confirmed by bulk RNA sequencing and immunofluorescence staining (Figures 4 and S4B), suggesting that myelination is still in an early phase. Nevertheless, spontaneous activity of cells in organoid slices was observed in calcium signal recordings (Figure S4C). Finally, ALI culture allowed us to achieve long-term culture beyond one year (Figure S5).

### Genetically modified cells of brain organoids display tumor-like properties

**Figure 5:**
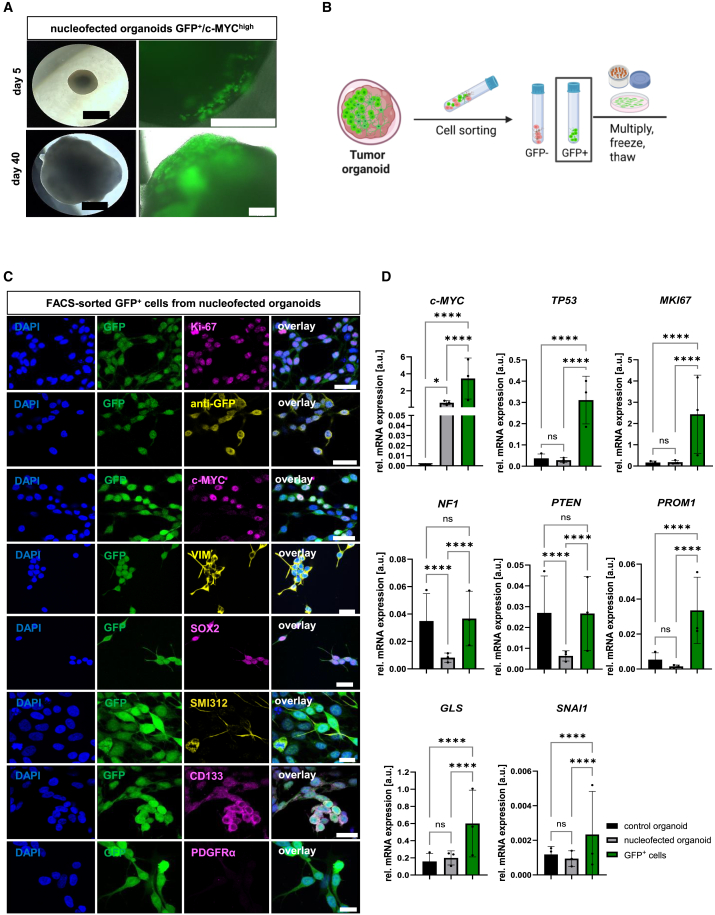
Genetically modified tumor-like cells generated in organoids show tumor-like properties (A) Representative microscopic images of organoids 5 and 40 days after nucleofection at day 11 of the culture. Scale bars: 930 μm left and 330 μm right. (B) GFP^+^/c-MYC^high^ cells isolated from nucleofected organoids based on the presence of GFP that can be further propagated as adherent cells or cryopreserved for later use. (C) Representative immunofluorescence staining of Ki-67 (magenta), anti-GFP (yellow), SOX2 (magenta), VIM (yellow), CD133 (magenta), c-MYC (magenta), PDGFRα (magenta), and SMI312 (yellow) for isolated GFP^+^ cells (green). Scale bars: 25 μm. (D) Relative mRNA expression of *c-MYC*, *TP53*, *MKI67*, *NF1*, *PTEN*, *PROM1*, *GLS*, and *SNAI1* in isolated GFP^+^ cells compared to control (sham-nucleofected) and nucleofected whole organoids at day 100 of the culture. Data are presented as mean ± SD for three independent experiments (*N* = 3) and three organoids per experiment (*n* = 3), ∗*p* < 0.05, ∗∗*p* < 0.01, ∗∗∗*p* < 0.001, ∗∗∗∗*p* < 0.0001. Statistical analysis was done using 2-way mixed ANOVA with Tukeýs post-test. See also Figures S6 and S7.

To generate a reproducible tumor model comprising genetically modified tumor-like cells fused as a tumor sphere with whole organoids (assembloid) or co-cultured with organoid slices, we first optimized the nucleofection procedure (including plasmid concentration and nucleofection program) (Figure S6). This was of crucial importance to preserve organoid integrity allowing for optimal propagation of tumor-like cells. Nucleofection of organoids with vectors carrying *Sleeping Beauty* transposase and *c-MYC* oncogene together with *GFP* (Figure S6A) resulted in the first appearance of GFP^+^ cells within 1 week after nucleofection, and in most cases their relatively rapid overgrowth of organoids as evidenced by the increase in GFP signal during the culture time (Figure 5A), which therefore was a limiting factor for a prolonged culture of nucleofected organoids. In addition, the randomness of nucleofection resulted in high variability of *MYC* overexpression observed as different GFP signal intensities between organoids (Figure S6E). To enhance the reproducibility of experiments, we isolated genetically modified cells from nucleofected organoids based on the presence of the co-expressed GFP, which was identified by FACS and confirmed by immunofluorescence (IF) staining (Figures 5B and 5C). The cells could be passaged and/or cryopreserved for expansion and/or later use. M-FISH analysis of 302 GFP^+^/c-MYC^high^ cells (*N* = 4, *n* = 67–97) compared to 198 control H9 cells (*N* = 3, *n* = 52–90) showed no clonal aberrations in either group (Figure S6F). As shown in Figure 5C, these GFP^+^/c-MYC^high^ cells exhibited high proliferative capacity (Ki-67^+^) and an immature phenotype (Sox2^+^), with the expression of an axonal proteins (marked with SMI312) to some extent and almost no detectable expression of the glial progenitor marker PDFGRα. In addition, they displayed invasive (VIM^+^) and migratory properties (scratch assay) as well as a (cancer)-stem-cell-like phenotype (CD133^+^) as shown in Figures 5C, S7B, and S7C. RT-qPCR analyses comparing the mRNA levels in non-nucleofected controls, nucleofected organoids, and isolated GFP^+^/c-MYC^high^ cells revealed a significant increase in the expression of *c-MYC* in nucleofected organoids and in isolated GFP^+^ cells (Figure 5D). GFP^+^ cells exhibited a significant increase in the expression of *TP53*, *MKI67*, *PROM1*, *GLS*, and *SNAI1* at mRNA level when compared to control organoids. However, there was no change in the expression of these markers between control and nucleofected organoids. Expression of the tumor suppressor markers *NF1* and *PTEN* was significantly decreased in nucleofected organoids when compared to control organoids and GFP^+^ cells. Conversely, GFP^+^ cells exhibited no significant difference in the expression of *NF1* and *PTEN* compared to control organoids (Figure 5D). Notably, certain proteins, such as c-MYC and GFP, exhibited considerable heterogeneity in the signal intensity observed in IF staining. GFP^+^ cells exhibited a strong albeit not significant decrease of neuronal and glial markers compared to the control organoids due to this heterogeneity (Figure S7).

### Cerebral organoids and organoid slices serve as scaffolds for autologous tumor-like cells

To increase comparability and reproducibility of the tumor model, GFP^+^/c-MYC^high^ cells were aggregated to form spheres and fused with normal sister organoids (assembloids).

**Figure 6:**
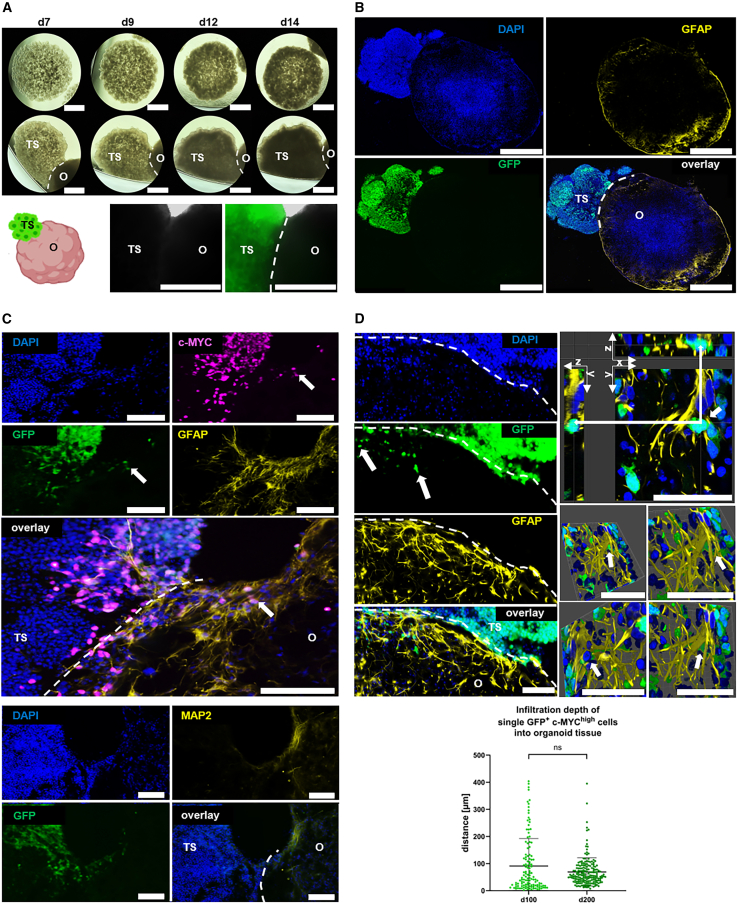
Genetically modified tumor-like cells assembled with whole organoids (A) Spheres generated using GFP^+^ tumor-like cells alone and after fusion with the whole organoid. The border area between the organoid and the fused tumor sphere is marked with a white dashed line. Scale bars: 1000 μm (upper panel), 330 µm (lower panel). (B) Representative immunofluorescence staining of GFAP (yellow) for the astrocytes of the organoid at day 100 of the culture fused with tumor-sphere with GFP^+^ cells (green) in 30 μm thick cryosection 14 days after fusion. The border area between the organoid and the fused tumor sphere is marked with a white dashed line. Scale bars: 1000 μm. (C) Representative immunofluorescence staining of c-MYC (magenta) for GFP^+^ cells (green) in the tumor sphere and of GFAP (yellow) or MAP2 (yellow) in the organoids at their interaction site in 30 μm thick cryosection 14 days after fusion of the tumor spheres with the organoids at day 100 of the culture. The border area between the organoid and the fused tumor sphere is marked with a white dashed line. Scale bars: 100 μm. (D) Representative immunofluorescence staining of GFAP (yellow) for the astrocytes of the organoid in the interaction with GFP^+^ cells (green) from the fused tumor sphere in the 3D image of 30 μm thick cryosection generated using z stack 14 days after fusion. The border area between the organoid and the fused tumor sphere is marked with a white dashed line. Scale bars: 50 μm (right side) and 100 μm (left side). Infiltration depth of GFP^+^/c-MYC^high^ cells measured as a distance from the interaction site with organoids at day 100 and day 200 of the culture 14 days after fusion. Data are presented as mean ± SD for three independent experiments (*N* = 3) and two to three organoids per experiment (n = 2–3), while each point represents the measurement of one single GFP^+^/c-MYC^high^ cell (125 cells at day 100 and 185 cells at day 200 of the culture). Statistical analysis was done using Mann Whitney test.

**Figure 7:**
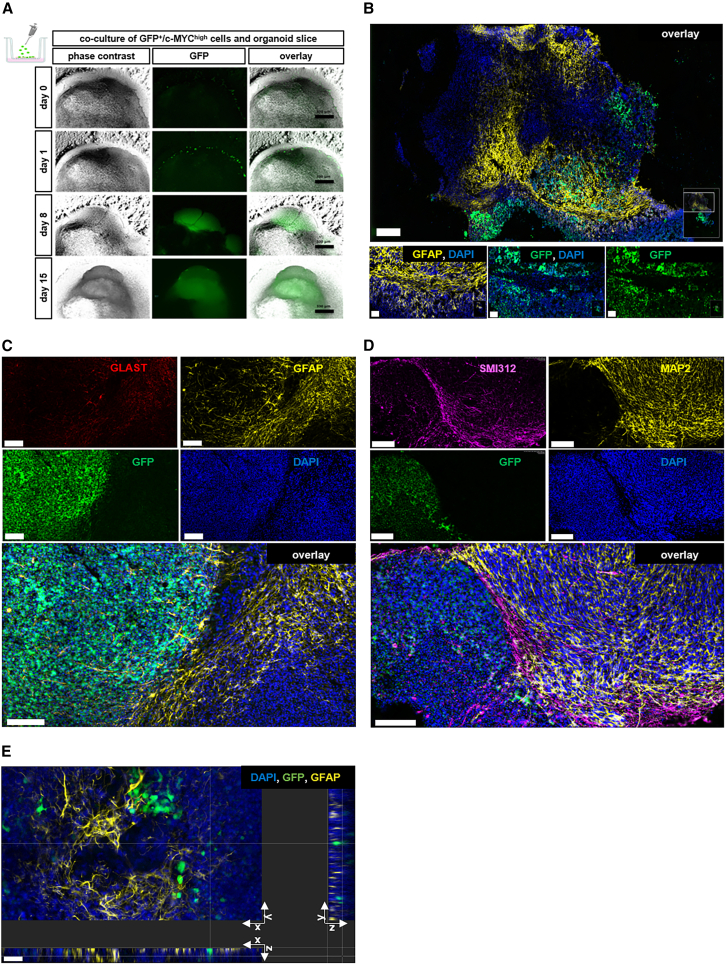
Genetically modified tumor-like cells cultured with organoids slices maintained at the air-liquid interface (A) Single tumor-like cells added on top of the organoid slice at ∼ day 50 of the culture show an increase in the GFP signal in time observed on day 0, day 1, day 8, and day 15 of the co-culture. Scale bars: 330 μm. (B) Representative immunofluorescence staining of GFAP (yellow) for the astrocytes of the organoid slice at day 100 of the culture in the interaction with GFP^+^ cells ∼50 days after addition. Scale bars: 300 μm. (C) Representative immunofluorescence staining of GLAST (red) and GFAP (yellow) in the organoid slice at day 100 of the culture containing tumor-like GFP^+^ (green) cells ∼50 days after addition. Scale bars: 100 μm. (D) Representative immunofluorescence staining of SMI312 (magenta) and MAP2 (yellow) in the organoid slice containing tumor-like GFP^+^ (green) cells. Scale bars: 30 μm. (E) Representative immunofluorescence staining of GFAP (yellow) for the astrocytes of the organoid slice at day 100 of the culture in the interaction with GFP^+^ cells (green) ∼50 days after addition in the 3D image of 30 μm thick cryosection generated using z stack. Scale bars: 30 μm.

**Figure 8:**
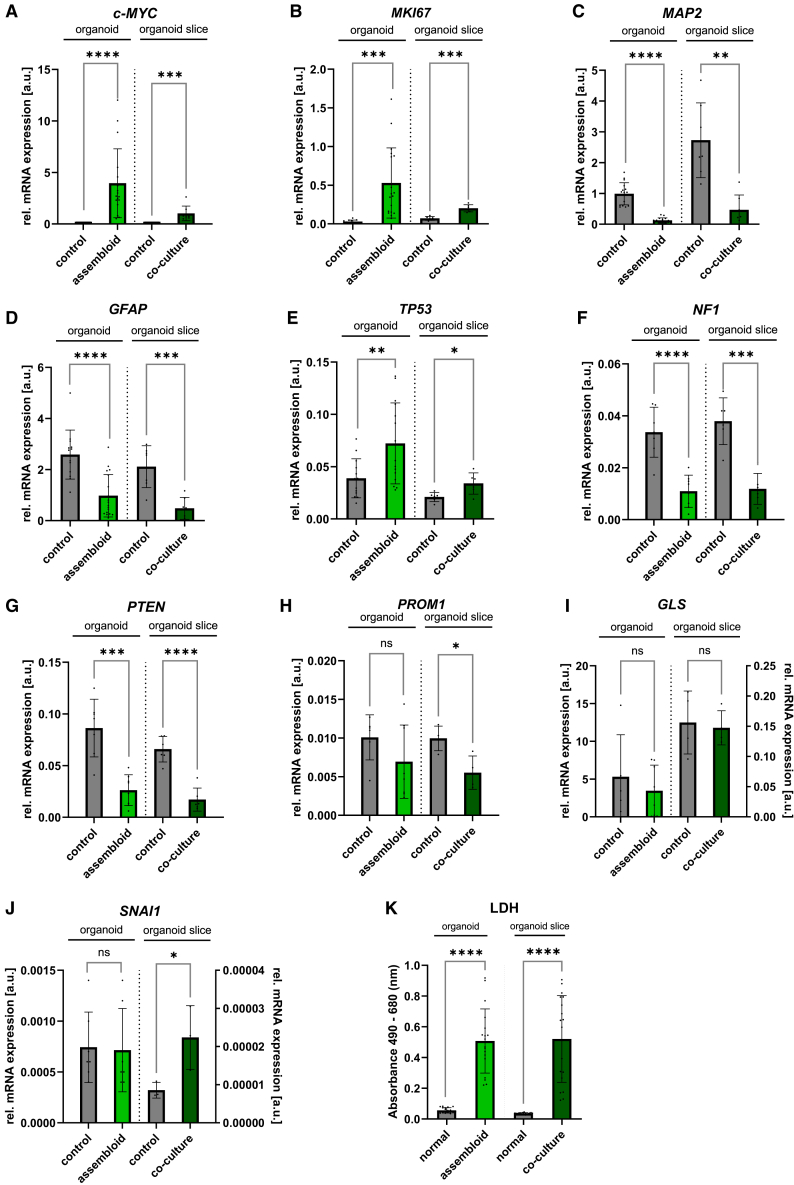
Tumor-like properties in whole organoids assembled with genetically modified tumor-like cells or in co-culture of organoid slices and tumor-like cells (A–J) Relative mRNA expression of *c-MYC*, *MKI67*, *MAP2*, *GFAP*, *TP53*, *NF1*, *PTEN*, *PROM1*, *GLS*, and *SNAI1* in the assembled whole organoids at day 100 of the culture 14 days after fusion with tumor spheres or co-cultured organoid slices at ∼ day 50 of the culture with tumor-like cells ∼50 days after addition compared to their respective controls (whole organoids or organoid slices at day 100 of the culture). Data are presented as mean ± SD for three to six independent experiments (N = 3–6) and two to three organoids per experiment (n = 2–3), ∗*p* < 0.05, ∗∗*p* < 0.01, ∗∗∗*p* < 0.001, ∗∗∗∗*p* < 0.0001. Statistical analysis was done using one-way ANOVA with Tukey’s post-test. (K) Lactate dehydrogenase (LDH) level measured in the co-culture of tumor-cells with whole organoids or organoid slices compared to their respective controls. Data are presented as mean ± SD for three to six independent experiments (N = 3–6) and 3 to 15 organoids per experiment (n = 3–15), ∗*p* < 0.05, ∗∗*p* < 0.01, ∗∗∗*p* < 0.001, ∗∗∗∗*p* < 0.0001. Statistical analysis was done using unpaired t test. See also Figure S8.

The tumor sphere part within the assembloids exhibited an increased cell density from day 9 after fusion compared to the tumor spheres cultured alone (Figure 6A). Through the fusion, the GFP^+^/c-MYC^high^ cells of the tumor sphere encountered contact to astrocytic cells (GFAP^+^) and neurons (MAP2^+^) from the outer edge of the organoid (Figures 6B and 6C). The protrusions of astrocytic cells (GFAP^+^) were observed to spread from the interaction site into the tumor sphere, while this was observed less extensively for the neurons (MAP2^+^) (Figure 6C). In turn, the GFP^+^ cells from the tumor sphere demonstrated the ability to infiltrate into the organoid (Figures 6C and 6D). The infiltrative potential of GFP^+^/c-MYC^high^ cells from the sphere was evident by the presence of GFP signal and IF staining in the organoid, particularly at sphere-organoid interface, with a small number of GFP^+^/c-MYC^high^ cells that were observed in deeper areas of the organoid (Figures 6C and 6D). Moreover, individual migrating GFP^+^ cells seemed to closely interact with astrocytic cells (GFAP^+^), which surrounded them with their protrusions (Figure 6D). In the co-culture of organoid slices and single tumor-like cells added on top of the slice, we observed an increase in GFP signal over time, resulting in overgrowth of the organoid slice (Figure 7A). GFP^+^/c-MYC^high^ cells were predominantly found near areas of the slice with increased GFAP and GLAST expression, although there was no or only little co-localization of the signals (Figures 7B and 7C). GFP^+^ cells, despite their proximity to areas with increased expression of neuronal markers (SMI312 and MAP2), were typically spatially segregated from those areas (Figure 7D). In addition, GFP^+^ cells were observed in deeper layers of organoid slices (Figure 7E), confirming their potential for infiltration. Cells in both tumor models retained proliferative and immature characteristics, as evidenced by *c-MYC* overexpression and increased *MKI67* expression, and decreased expression of *MAP2* and *GFAP* compared to their respective controls (Figures 8A–8D). The expression of *TP53* was higher in the co-culture than in the control (Figure 8E), while the expression of *NF1* and *PTEN* was decreased in both tumor models compared to normal organoids or slices (Figures 8F–8G). This reflects the situation in the whole nucleofected organoids (Figure 8). In contrast to its expression in GFP^+^ cells, *PROM1* showed a decreased expression in the co-culture compared to the control (Figure 8H). There was no discernible difference in the expression of *GLS* between the control and co-culture, while the expression of *SNAI1*, a master regulator of epithelial to mesenchymal transition (EMT),^32^ was increased in the co-culture, but not in the assembloids (Figures 8I and 8J). Bulk RNA sequencing data (Figure S8) revealed that whole organoids and organoid slices clustered far away from tumor cells and spheres, while assembloids represented the third cluster in between the two (Figure S8A). This is reflected in the 50 most differentially expressed RNAs (Figure S8B). Bulk RNA sequencing also showed the expression of additional factors that support tumor cell survival, invasion, and EMT in our model system. For instance, *RELN*, a key regulator of neuronal migration, was predominantly expressed in normal brain slices and organoids, but was largely absent in co-cultures, similarly other members of the Reelin signaling axis including to *DAB1*, *VLDRL*, and *SRC* (Figure S8C). *SERPINE1*, a known pro-survival factor, was expressed in both, control organoids and assembloids, and to a lesser extend in organoid slices, but was notably undetectable in tumor spheres and isolated tumor-like cells (Figure S8). Brain-derived neurotrophic factor (BDNF), which promotes neuronal maturation and plasticity, was expressed in whole organoids and organoid slices, with reduced levels observed in assembloids with lower expression in tumor-like cells and tumor spheres. In contrast, *NGF*, associated with pro-tumoral effects, was expressed in tumor spheres and tumor-like cells, with lower expression in assembloids. While tumor-like cells showed low *SRC*, *SRCIN1*, and *DAB2IP* expression, *SLA* was predominantly expressed in these cells. Metalloproteinase ADAM15 and MMP2 were expressed in tumor-like cells and tumor spheres to some extent but notably absent in normal organoids and organoid slices (Figure S8C). Both tumor models showed a significant increase in cell death/necrosis-associated LDH level in the cell culture medium compared to their respective controls (Figure 8K).

### Responses of cerebral organoids and organoid slices to X-ray irradiation

Given the widespread presence of c-MYC in various brain tumor types and its role in mediating responses to therapeutic interventions, including conventional X-ray radiotherapy, we conducted preliminary irradiation experiments to assess the behavior of the tumor models based on GFP^+^/c-MYC^high^ tumor-like cells—generated using the two different culture methods described above (Figure 1).

**Figure 9:**
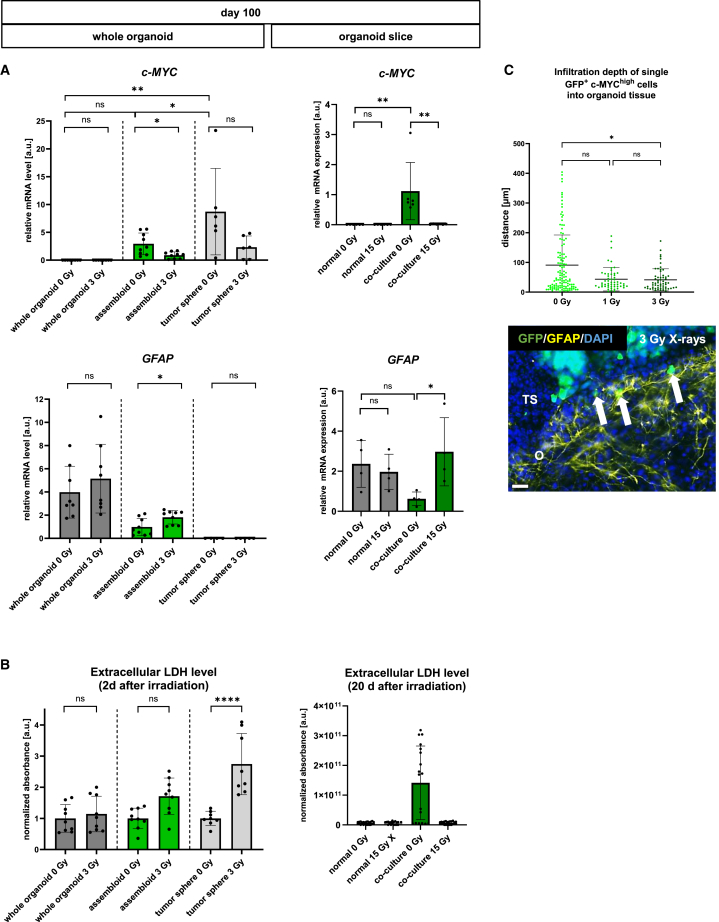
Response of whole organoids assembled with genetically modified tumor-like cells or in co-culture of organoid slices and tumor-like cells to different doses of X-rays (A) Relative mRNA expression of *c-MYC* and *GFAP* in whole organoids, whole organoids assembled with tumor spheres, and tumor spheres 7 days after irradiation with 0 Gy or 3 Gy X-rays, which was performed 7 days after fusion. Relative mRNA expression of *c-MYC* and *GFAP* in normal organoid slices and in organoid slices co-cultured with GFP^+^/c-MYC^high^ tumor-like cells 20 days after irradiation. Data are presented as mean ± SD for three to six independent experiments (N = 3–6) and one to three samples per experiment (n = 1–3), ∗*p* < 0.05, ∗∗*p* < 0.01, ∗∗∗*p* < 0.001, ∗∗∗∗*p* < 0.0001. Statistical analysis was done using one-way ANOVA with Tukey’s post-test for assembloids and one-way ANOVA with Šídák’s post-test for organoid slices. (B) Extracellular lactate dehydrogenase (LDH) level of whole organoids at day 100 of the culture, whole organoids at day 100 of the culture assembled with tumor spheres, and tumor spheres 2 days after irradiation with 0 Gy, or 3 Gy X-rays, which was performed 7 days after fusion. Extracellular LDH level of normal organoid slices, and organoid slices co-cultured with GFP^+^/c-MYC^high^ tumor-like cells 20 days after irradiation. Data are presented as mean ± SD for three to six independent experiments (N = 3–6) and 3 samples per experiment (*n* = 3), ∗*p* < 0.05, ∗∗*p* < 0.01, ∗∗∗*p* < 0.001, ∗∗∗∗*p* < 0.0001. Statistical analysis was done using one-way ANOVA with Tukey’s post-test for assembloids and one-way ANOVA with Šídák’s post-test for organoid slices. (C) Infiltration depth of GFP^+^/c-MYC^high^ cells measured as a distance from the interaction site with organoids at day 100 of the culture 7 days after irradiation with 0 Gy, 1 Gy, or 3 Gy X-rays, which was performed 7 days after fusion. Data are presented as mean ± SD for three independent experiments (*N* = 3) and one to three assembloids per experiment (n = 1–3), while each point represents the measurement of one single GFP^+^ c-MYC^high^ cell (125 cells after 0 Gy, 54 cells after 1 Gy, and 58 cells after 3 Gy). Statistical analysis was done using Kruskal-Wallis test and Dunn’s post-test. Representative immunofluorescence staining of GFAP (yellow) for the astrocytes of the organoid in the interaction with GFP^+^ cells (green) from the fused tumor sphere in the 3D image of 30 μm thick cryosection generated using z stack 7 days after irradiation with 3 Gy X-rays, which was performed 7 days after fusion. The border area between the organoid and the fused tumor sphere is marked with a white dashed line. White arrows mark single GFP^+^ cells that infiltrated into organoid at depth of 1–10 μm. Scale bars: 10 μm.

*C-MYC* mRNA was hardly detectable in cerebral organoids and organoid slices, but highly expressed in tumor spheres, assembloids of normal organoids with tumor spheres, and organoid slices co-cultured with GFP^+^/c-MYC^high^ tumor-like cells. Irradiation led to a significant decrease in the c-*MYC* mRNA level in assembloids subjected to 3 Gy X-rays 7 days post-irradiation and in the co-culture of organoid slices and tumor-like cells exposed to 15 Gy X-rays 20 days post-irradiation when compared to the sham-irradiated controls (Figure 9A). After irradiation, *GFAP* mRNA levels remained unchanged in whole organoids or organoid slices. However, in comparison to sham-irradiated controls, 3 Gy or 15 Gy X-ray irradiation led to a significant increase in *GFAP* mRNA expression in assembloids or co-cultures of organoid slices with GFP^+^/c-MYC^high^ tumor-like cells, respectively, when compared to the corresponding sham-irradiated controls (Figure 9A).

Contrary to the expectation that irradiation leads to an increased cell death/necrosis,^33^ we observed no significant changes in the level of extracellular LDH as an indication for radiation-induced necrosis 2 days post-irradiation in whole organoids or normal organoid slices 20 days post-irradiation. Tumor spheres responded to irradiation with an acute (2 days) increased extracellular LDH level, while co-cultures 20 days post-irradiation showed a decreased LDH release compared to their respective sham-irradiated controls (Figure 9B).

Infiltration depth of GFP^+^/c-MYC^high^ tumor-like cells into the organoid part of assembloids was significantly decreased 7 days after irradiation with 3 Gy X-rays (Figure 9C). Particularly, the cells migrating deep into the organoid tissue observed in the sham controls were absent in the irradiated samples.

## Discussion

The field of organoid research is advancing rapidly, with a growing body of protocols and data enabling continuous improvement of model systems to study organ development and disease. Human cerebral organoids, for instance, have been instrumental in advancing research on brain development, neurological and neurodegenerative diseases, and cancer,^21^^,^^34^ while circumventing challenges related to the availability of human tissue and reducing the reliance on animal models. The involvement of various components of the microenvironment, including immune cells, fibroblasts, and stroma, in tumor progression is well documented.^35^ However, the role of glia cells in brain tumors has not been extensively studied, despite their critical functions in supporting other cell types, e.g., through the activation of proMMP2 and the expression of AEG-1, both implicated in glioma invasion.^36^^,^^37^

We adopted a previously published protocol for the differentiation of pluripotent stem cells into cerebral organoids^24^ with minor modifications. This protocol promotes the differentiation of glial cells, including oligodendrocytes and astrocytes. Organoids generated according to the Marton protocol^24^ lack spontaneous organization, and contain only few proliferation zones, which are specific for unguided differentiation protocols that are based on self-patterning and spontaneous differentiation with minimal usage of external patterning factors (see in the study by Paşca et al.^38^ for definition), e.g., by Lancaster et al.^23^ Nevertheless, the complex cellular and structural composition, which is an important requirement for a 3D model system to be characterized as an organoid, was met as evidenced by immunofluorescence staining and qPCR analyses. In addition, spontaneous cell activity (calcium signals) was measured in organoid slices, but could not be detected in whole organoids, due to technical limitations of the experimental setup. However, the expression of pre- and postsynaptic markers, such as VAMP2 and HOMER or more specific markers of excitatory synapses, such as SYN1 and PSD95, which are known to play key roles in synaptic signaling and plasticity,^39^^,^^40^ in close proximity within whole organoids, serves as an indicator of functional connectivity.

Previous studies showed that whole organoids are capable of spontaneous activity.^41^ To increase and harmonize neuronal organization, we facilitated neural tube formation and specification via the use of microfilaments and Matrigel embedding as described by Lancaster et al.^23^ before using guided differentiation according to Marton et al. to induce glial progenies.^24^ This approach facilitated a bi-phasic differentiation process. The first phase, lasting approximately 50 days, was critical for cell proliferation, resulting in an increase in organoid size to approximately 3–4 mm in diameter, consistent with previous reports.^42^ Beyond day 50, organoid growth plateaued, and this was corroborated by low number of Ki-67^+^ cells observed at day 100. In the second phase, maturation of cells occurred, as evidenced by immunofluorescence staining of neuronal and glial markers, such as the intermediate filament staining SMI312, GFAP, and MBP. Notably, the organoids also retained progenitor cells, including those expressing nestin. Nestin, an intermediate filament protein typically expressed by neural progenitor cells, is re-expressed in reactive astrocytes in the injured brain.^43^ The neuronal marker MAP2 was detected in organoids at an early stage of differentiation (cells were MAP2^+^ and DCX^+^), and not only in more mature neurons as usually described for MAP2.^44^ DCX, which regulates microtubule dynamics and SMI312, marking intermediate filaments supporting the axonal structure, were also present in less mature cells and more differentiated neurons. The gap junction protein CX43 that is important for intercellular communication during neural development and in astrocytes further validated the maturation process.^45^ The shift in marker expression and the reorganization of cellular composition reflect the differentiation and maturation of the organoids as corroborated by single nuclei sequencing analysis. It shows the expression of progenitor markers, as well as mature neuronal and glia markers expressed by different cell subpopulations. Some of these markers are related not only to maturation but also to cell functionality. The minimal number of caspase-3 positive cells suggested that the organoid models remained viable throughout the culture period. However, as organoid size increases, supply with oxygen and nutrients are aggravated leading to a so-called necrotic core. Cell death in the organoid interior was confirmed by shrunken and fragmented nuclei and an increased level of extracellularly released LDH.^46^ To avoid this, we employed the ALI technique for culturing organoid slices. Originally described for hippocampal slices^47^ and later applied to cerebral organoids generated by unguided differentiation,^26^ the ALI technique improved oxygen and nutrient supply, reducing cell death and enabling culture maintenance for over one year. The enhanced nutrient and oxygen delivery resulted in better differentiation and maturation, particularly evidenced by the development of robust axonal networks (SMI312 staining) extending from organoid slices cultured on porous membrane inserts. These axonal tracts, which resembled subcortical projection tracts,^23^^,^^26^ were more pronounced and organized than those observed in whole organoids. While astrocytic markers such as GLAST, CX43, and GFAP showed similar expression patterns in both organoids and organoid slices, lower mRNA expression of the immature astrocyte marker *VIM* and higher expression of the neural marker (*MAP2*) in organoid slices indicated a more advanced maturation of neural cells. Additionally, PDGFRα signaling, known to regulate oligodendrocyte precursor cell numbers,^48^ was more prominently expressed in organoid slices compared to whole organoids. This further corroborates the advanced differentiation of oligodendrocyte precursors and the presence of MBP, although at low levels in both mRNA and protein analyses, indicates that myelination at day 100 of the culture is still in its infancy. This in accordance with single nuclei RNAseq data, identifying 7% and 10% of the cells as mature oligodendrocytes in whole organoids and organoid slices, respectively. In conclusion, the generated cerebral organoid models faithfully recapitulate key features of brain development, including the complex cellular composition of the cortex. With a high abundance of astrocytes, these models provide a solid foundation for investigating the role of glial cells in brain tumors, enabling closer examination of their involvement in tumorigenesis.

Much effort has been employed to establish *in vitro* tumor models, particularly for brain tumors, with most culture methods aiming to preserve the characteristics of the parental tumor (reviewed in Ledur et al.^49^) albeit with varying outcomes and limited scalability.^50^^,^^51^ The advent of organoid technologies has enabled the co-culture of primary tumor tissue with pluripotent stem cell-derived cerebral organoids.^52^^,^^53^ A recent study showed that tumor medulloblastoma cell lines co-cultured as spheroids with cerebral organoids retained their malignant properties.^54^ However, such approaches raise concerns regarding the mixing of distinct genetic backgrounds and developmental stages, as adult tumor tissue or cell lines are combined with early embryonic cerebral organoids. This issue has been addressed through clonal mutagenesis, specifically the amplification of oncogenes and the suppression of tumor-suppressor genes, to induce tumors in brain organoids.^21^^,^^22^ In this study, we used a modified version of one of such protocols, where the *c-MYC* oncogene was overexpressed in cerebral organoids using the *Sleeping Beauty* transposon system.^22^ The *c-MYC* oncogene is variably expressed across different brain tumors types, e.g., in about 70% of glioma, including low and high grade tumors,^55^ and is amplified in up to 17% of group 3 medulloblastomas, the most common malignant brain tumor in children.^56^ Given that nucleofection of cells in organoids is a random process,^22^ it leads to a heterogeneous distribution of genetically modified cells, which can overtake the entire organoid, thereby compromising reproducibility and limiting suitability for long-term studies. Thus, we addressed this variability by isolating GFP^+^/c-MYC^high^ cells from nucleofected organoids via FACS. These isolated cells could be passaged and/or cryopreserved, enabling high-throughput scaling and usage at a self-determined time. Furthermore, these genetically modified cells were cultured in aggregates and fused to sister organoids or co-cultured with organoid slices, enhancing the reproducibility of the model and ensuring a consistent genetic background (i.e., autologous setting) across tumor-like and normal cells. The specificity of co-expression of GFP and c-MYC were confirmed by immunofluorescence (IF) staining. GFP^+^/c-MYC^high^ cells displayed tumor-like properties, including high proliferation (Ki-67 expression), an immature and stem cell-like phenotype (SOX and CD133 expression), and invasive potential (VIM expression) and in 2D culture. Axon marking SMI312 was detected in some cells, while glial progenitor marker PDFGRα was scarcely expressed, suggesting a neuronal rather than a glia lineage commitment of these cells. Additionally, the transcriptional profile of GFP^+^/c-MYC^high^ cells differed significantly from that of control cells in sister organoids, which lacked detectable *c-MYC* expression. Notably, GFP^+^/c-MYC^high^ cells exhibited elevated expression of *TP53*, *GLS*, and *SNAI1* compared to cells of control organoids, suggesting altered bioenergetics and mesenchymal features commonly associated with tumorigenesis,^57^^,^^58^ while retaining *TP53* regulatory mechanisms. This heterogeneity of *c-MYC* expression within the cell population and between batches mirrors the variability observed in human tumors,^59^ highlighting the challenges of tumor modeling. In nucleofected organoids, where normal cells still predominate, this variability resulted in no significant changes or decreased expression of *TP53*, *MKI67*, *PROM1*, *GLS*, and *SNAI1* when compared to GFP^+^/c-MYC^high^ cells. As previously described, *TP53* is a well-known transcriptional target of c-MYC, with both c-MYC and E1A shown to stabilize *TP53* and trigger *TP53*-dependent transcription.^60^^,^^61^ In our system, the expression of *c-MYC* was positively correlated with *TP53* and *MKI67* expression, consistent with its role as a prognostic biomarker in brain tumors such as gliomas and glioblastomas.^62^^,^^63^ As anticipated, the expression of tumor suppressor genes *NF1* and *PTEN* was reduced in nucleofected organoids compared to control organoids. However, no difference in *NF1* and *PTEN* expression between GFP^+^/c-MYC^high^ cells and control organoids suggests that these genes may be regulated by factors other than *c-MYC* in our model system.^64^^,^^65^ It has been reported that *PTEN* can acquire a pro-tumoral role by stabilizing gain-of-function *TP53* mutants in glioma cells.^66^^,^^67^^,^^68^ Furthermore, *PROM1*, a marker associated with tumor stemness and glioblastoma progression, showed a positive correlation with *MKI67* in some tumor models.^69^ In our study, *PROM1* expression was higher in tumor-like cells compared to control organoids. However, in tumor assembloids and in co-culture with organoid slices, *PROM1* expression was lower than in the normal organoids or organoid slices. As *PROM1* expression in tumors is altered by autophagy factors or lactate,^70^^,^^71^ these could be involved in *PROM1* alterations seen here.

The tumor-like properties of GFP^+^/c-MYC^high^ cells were further confirmed in our tumor models, where the GFP signal increased over time and GFP^+^ cells were detected in organoids or organoid slices, reflecting the proliferative and invasive potential of these cells. *C-MYC* overexpression has been shown to contribute significantly to the aggressiveness of medulloblastoma,^72^^,^^73^ although the effects of exogenous *c-MYC* expression via the *Sleeping Beauty* transposon system may differ from endogenous *c-MYC* in patient tumors. In a study employing a similar co-culture approach with medulloblastoma cell lines and cerebral organoids, *NEUROD1*—a key molecular determinant of granule cell identity—was expressed only in the co-culture, which represents a more physiological condition, but not in cell lines or tumor spheres alone.^54^ In our model, we detected *NEUROD1* expression in GFP^+^/c-MYC^high^ cells, tumor spheres, and the assembloids, but not in whole organoids or organoid slices. This supports the hypothesis of the presence of a cancer stem cell-like state, reminiscent of those seen in medulloblastoma.

Mesenchymal features of the tumor model were corroborated by the expression of *SNAI1*.^57^ No significant changes in *GLS* expression between tumor models and controls suggest that glutamine metabolism, a key feature in tumor progression, remains unaltered in our system.^74^ Additionally, an increase in extracellular LDH levels in co-cultures may be associated with the massive proliferation and overgrowth of GFP^+^/c-MYC^high^ cells and the associated increased competition for space and nutrients, a hallmark of many brain tumors.^75^^,^^76^ The gene expression profiles obtained from nucleofected whole organoids and co-cultured systems were highly consistent, demonstrating the robustness of the co-culture model in providing more reliable and physiologically relevant results compared to traditional 2D cultures. Moreover, this approach allows for experiments at defined and later time points, including long-term culture, which is not feasible with random nucleofection without FACS sorting.

Genetically modified GFP^+^/c-MYC^high^ cells in spheres benefited from the cultivation with normal organoids, exhibiting increased cell density compared to spheres alone. Normal organoids provided not only a larger surface for tumor-like cells to spread but also extended GFAP-positive astrocytic protrusions into the sphere. This supports the known role of astrocytes and oligodendrocytes in tumor progression and invasion through secretion of various factors.^77^^,^^78^ Tumor cells can also activate astrocytes via the JAK/STAT pathway, promoting an immunosuppressive environment.^79^ Brain metastases of small cell lung cancer (SCLC) have been shown to secrete REELIN (*RELN*), which recruits reactive astrocytes and activates developmental programs in these cells.^80^ The expression of *RELN*, a regulator of neuronal migration,^81^ in normal brain slices and organoids, but not in co-cultures suggests that *RELN* expression may be modulated or downregulated in the tumor microenvironment, possibly after initial astrocyte recruitment has occurred. These astrocytes, once recruited, not only can localize to the metastatic niche but can also promote tumor growth through the secretion of pro-survival neuronal factors such as SERPINE1.^80^ The bulk RNA-seq data show that *LRP8* (*APOER2*), *CRK*, *PI3K* were similarly expressed across all models as a part of core signal transduction modules, and the expression of *RELN*, *DAB1*, *VLDR*, and *SRC* predominantly by normal organoids and organoid slices implies the involvement of the Reelin signaling axis in astrocytes recruitment. *SERPINE1*, a pro-survival factor, was expressed in both control organoids and assembloids, but notably absent in tumor spheres and tumor-like cells alone, suggesting that this pro-tumorigenic factor is astrocyte-derived in the co-culture context. In addition, astrocytes are known to secrete NGF and BDNF (reviewed in the study by Albini et al.^82^) in response to injury or neuronal activity, and these neurotrophins have been shown to support tumor cell survival, invasion, and EMT in several cancers, including head and neck squamous cell carcinoma, and glioma.^83^^,^^84^ *NGF*, and *NEUROD1* expression in tumor spheres and tumor-like cells suggests autocrine signaling aiding tumor survival, while *BDNF* expression in normal slices suggest potential paracrine signaling mechanisms that may contribute to tumor cell migration and survival. While downregulation of *SRCIN1*, *DAB2IP*, and *SRC* expression in tumor-like cells and tumor spheres indicates a loss of regulatory signaling, possibly contributing to malignancy, SLA might compensate these regulators, and the upregulation of matrix metalloproteinases MMP13 and ADAM15, known to contribute to glioma/glioblastoma aggressiveness,^85^^,^^86^ supports the invasive potential of these cells. The involvement of astrocytes in the tumor progression is further corroborated by previous findings in glioblastoma, where tumor cells formed gap junctions with astrocytes, allowing direct cellular communication that supports tumor invasion.^87^ Although the presence of these factors suggests the immediate interaction between tumor cells and astrocytes and the activation of developmental programs in these cells, one has to consider that the Reelin pathway is involved in normal neuronal development (as reviewed in the study by Jossin et al^88^) and therefore may be highly expressed in the organoids and organoid slices compared to the immature tumorous cells.

Interestingly, while neuronal cells showing axonal structures (SMI312) and MAP2 expression were present in our model, they were spatially segregated from GFP^+^ cells and appeared less permissive to tumor cell invasion, a finding consistent with xenograft models in which glioblastoma cells predominantly invade astrocyte- and microglia-rich regions rather than neuronal areas.^89^ Despite this, a small proportion of GFP^+^/c-MYC^high^ cells were able to migrate into normal organoid tissue or infiltrate organoid slices, resembling the behavior of disseminated tumor cells in glioblastomas.^90^ Although GFP^+^/c-MYC^high^ cells were evenly distributed over the organoid slice at the beginning of the co-culture, larger aggregates, rather than evenly distributed cells, were observed at later time points. Given that c-MYC is a key role in clonal complexity and cell-cell competition in tumors (^91^; reviewed in the study by Madan et al.^92^), a clonal expansion of GFP^+^/c-MYC^high^ cells is a plausible outcome and could further explain larger dominant aggregates of these cells in the co-culture with organoid slices.

While GFAP, a marker highly expressed in astrocytomas, showed minimal expression in highly proliferative GFP^+^ cells, it does not correlate directly with malignancy grade, as different GFAP isoforms can coexist in these tumors.^93^ However, c-*MYC* overexpression alone is insufficient to induce a glioblastoma-specific phenotype. Future improvements to this model will involve introducing deletions in tumor suppressor genes commonly affected in pediatric glioblastoma and conducting single-cell analyses to further refine the model. The 3D microenvironment of organoids or organoid slices, which exhibit key features of the brain, including a high proportion of glial cells—particularly astrocytes—provides a supportive framework for the proliferation, migration, and invasion of genetically modified cells with tumor-like characteristics. Significant cell killing and impaired tissue infiltration of tumor-like cells upon irradiation with X-rays, as one treatment approach to fight brain tumors, reflects what is seen *in vivo*. Thus, we have developed a robust and reproducible autologous model system with adaptable tumor initiation step, i.e., introduction of other oncogenes and tumor suppressor genes, utilizing two distinct culture approaches, enabling the long-term study of various treatment modalities, such as radiotherapy, chemotherapy, and immunotherapy, in addition to studies about the precise interaction of tumorous and normal cells at the tumor margin in response to the treatment. This approach ensures that all cells originate from the same genetic background, offering a more accurate and personalized platform for therapeutic evaluation.

### Limitations of the study

The approach presented in this study underscores the usefulness of human brain organoids and the versatility of the tumor model. It can be adapted not only to study a vast number of oncogenes and tumor suppressor genes involved in brain cancer initiation and progression, but also to study the impact of tumorous tissue on certain brain regions by using region-specific organoids instead of cerebral ones.

However, the model implies certain limitations that are intrinsic for many studies regarding the use of brain organoids: despite morphogenetic intervention resulting in a physiological number of astrocytes in the organoids, the number of oligodendrocytes in the presented tumor model was rather low and myelination still in its infancy. This issue could be overcome by applying protocol alterations as suggested by Yan et al.^94^ In addition, the system still lacks the necessary components important to evaluate aspects involving vasculature and the brains intrinsic immune system, the microglia. This issue has been addressed by an *in vivo* xenotransplantation approach resulting in functionally mature human microglia operating within a vascularized immunocompetent environment.^95^ Yet, this tactic thwarts the scalability of the organoid system and its advantage to help minimizing animal experimentations. Therefore, others attempted to vascularize brain organoids by recapitulating neural tube angiogenesis or generating organoids from a mix of endothelial and stem cells (reviewed in the study by Kistemaker et al.^96^). Introducing said cell types would improve the tumor model and support its translational impact.

## Resource availability

### Lead contact

Further information and requests for resources and reagents should be directed to the lead contact, Insa S. Schroeder (i.schroeder@gsi.de).

### Materials availability

All materials needed to evaluate the conclusions in the paper are present in the paper and/or the supplemental information. This study did not generate new unique reagents.

### Data and code availability

Raw data are uploaded on open access repository Zenodo (Zenodo: https://doi.org/10.5281/zenodo.15525634). The raw bulk RNA-seq and single nuclei RNA-seq data, as well as corresponding processed data reported in this paper have been deposited in the Gene Expression Omnibus (GEO) repository (https://www.ncbi.nlm.nih.gov/geo/) as GEO: GSE301276 (RNA-seq) and GEO: GSE301277 (snRNA-seq). This paper does not report original code. All other data, such as microscopy images, are available from the corresponding author on reasonable request.

## Acknowledgments

The authors would thank members of stem cell differentiation and cytogenetics group led by I. Schroeder for helpful discussions and valuable suggestions and particularly to Emilia Choman, Dr. Carola Hartel, and Jennifer Persigehl for excellent technical assistance. We thank Daniela Di Giovanni for her invaluable support in the establishment and validation of synaptic marker staining protocols during her master’s thesis. We further thank Estrella Passerat de la Chapelle, Elena Meier, and Kim Knorr for their support with preparation of samples and immunofluorescence stainings during their masters theses. This work is supported by the 10.13039/501100002347German Federal Ministry of Research, Technology and Space (02NUK049A and 02NUK081A) and the 10.13039/100000002NIH grant 1RO1CA256848-01. The publication is funded by the Open Access Publishing Fund of GSI Helmholtzzentrum fuer Schwerionenforschung.

## Author contributions

T.B. and E.S. planned and performed experiments, analyzed data, interpreted results, and wrote the manuscript. L.K. performed confocal imaging and contributed to the interpretation of results. S.H. planned and performed Ca imaging experiments and analyzed data. M.M. and C.T. supervised electrophysiology experiments and interpreted data. D.R.G. and I.S.S. conceived and supervised the project and data interpretation.

## Declaration of interests

The authors declare no competing interests.

## STAR★Methods

### Key resources table

**Table:** 

REAGENT or RESOURCE	SOURCE	IDENTIFIER
**Antibodies**		

SOX2 (rabbit)	Thermo Fisher Scientific	Cat# A24339; RRID: AB_2924437
Nestin (mouse)	BD Biosciences	Cat# 611658; RRID: AB_399176
DCX (rabbit)	Abcam	Cat# ab18723; RRID: AB_732011
CX43 (rabbit)	Abcam	Cat# ab11370; RRID: AB_297976
SMI312 (mouse)	BioLegend	Cat# 837904; RRID: AB_2566782
GFP (chicken)	Thermo Fisher Scientific	Cat# A10262; RRID: AB_2534023
VIM (mouse)	Thermo Fisher Scientific	Cat# 14-9897-82; RRID: AB_10597910
CD133 (rabbit)	Abcam	Cat# ab19898; RRID: AB_470302
c-Myc (mouse)	Thermo Fisher Scientific	Cat# MA1-980; RRID: AB_558470
c-Myc (rabbit)	Cell Signaling Technology	Cat# 13987; RRID: AB_2631168
Ki-67 (rabbit)	Abcam	Cat# ab16667; RRID: AB_302459
PDGFRα (rabbit)	Atlas Antibodies	Cat# HPA004947; RRID: AB_2732399
GLAST (rabbit)	Thermo Fisher Scientific	Cat# PA5-111080; RRID: AB_2856490
MBP (rabbit)	Abcam	Cat# ab218011; RRID: AB_2895537
SYN1 (rabbit)	Synaptic Systems	Cat# 106103; RRID: AB_11042000
VAMP2 (rabbit)	Synaptic Systems	Cat# 104202; RRID: AB_887810
HOMER1 (mouse)	Synaptic Systems	Cat# 160011; RRID: AB_2120992
PSD95 (mouse)	Synaptic Systems	Cat# 124 011; RRID: AB_10804286
MAP2 (rabbit)	Abcam	Cat# ab225315; RRID: AB_3517252
GFAP (rabbit)	Abcam	Cat# ab194325; RRID: AB_3662092
Caspase-3 active (rabbit)	GeneTex	Cat# GTX22302; RRID: AB_384753
AF488 donkey anti-mouse	Thermo Fisher Scientific	Cat# A24350; RRID: AB_2924437
AF594 donkey anti-rabbit	Thermo Fisher Scientific	Cat# A24343; RRID: AB_2924437
AF568 goat anti-mouse	Thermo Fisher Scientific	Cat# A11004; RRID: AB_2534072
AF594 goat anti-rabbit	Thermo Fisher Scientific	Cat# A11012; RRID: AB_2534079
AF594 goat anti-mouse	Thermo Fisher Scientific	Cat# A21235; RRID: AB_2535804
AF594 donkey anti-mouse	Thermo Fisher Scientific	Cat# A32744; RRID: AB_2762826
AF647 goat anti-chicken	Thermo Fisher Scientific	Cat# A32933; RRID: AB_2762845
AF647 goat anti-mouse	Thermo Fisher Scientific	Cat# A21235; RRID: AB_2535804

**Chemicals, peptides, and recombinant proteins**		

Laminin-521	BioLamina	Cat# 600962
mTeSR™1 basal medium medium	STEMCELL Technologies	Cat# 85851
mTeSR™1 5X supplement	STEMCELL Technologies	Cat# 85852
Penicillin and streptomycin	Merck	Cat# A2212
TeSR™-E8™ basal medium	STEMCELL Technologies	Cat# 05991
TeSR™-E8™ 25X supplement	STEMCELL Technologies	Cat# 05992
ReleSR	STEMCELL Technologies	Cat# 05872
DMEM+GlutaMAX™-1 with 4.5 g/L D-Glucose and Pyruvate	Gibco	Cat# 31966–021
Fetal bovine serum superior	Sigma-Aldrich	Cat# S0615
Rho-associated protein kinase inhibitor	Tocris Bioscience	Cat# 1254
Anti-adherence rinsing solution	STEMCELL Technologies	Cat# 07010
VICRYL™ surgical sutures, violet, absorbable	Johnson & Johnson International	Cat# V632H
Polyvinyl alcohol (PVA)	Sigma-Aldrich	Cat# 363170
Essential 6™ Medium	Gibco	Cat# A1516401
Dorsomorphin	Biogems	Cat# 8666430
SB-431542	Torcris	Cat# 1614
Dulbecco’s Modified Eagle Medium/Nutrient Mixture F-12 (DMEM/F12) with L-Glutamine and 15 mM HEPES	Gibco	Cat# 31330–038
Neurobasal® medium without L-Glutamine	Gibco	Cat# 21103–049
*N*-2 supplement (100X)	Gibco	Cat# 17502–048
B27® supplement without vitamin A (50X)	Gibco	Cat# 12587–010
Insulin solution human	Sigma	Cat# 19278
β-mercaptoethanol	Sigma	Cat# M6250
GlutaMAX™-1 (100X)	Gibco	Cat# 35050–038
Minimum Essential Medium – Non-essential amino acid solution (MEM-NEAA) (100X)	Gibco	Cat# 11140–035
Recombinant human epidermal growth factor	PeproTech	Cat# AF-100-15
Recombinant human basic fibroblast growth factor	PeproTech	Cat# 100-18b
Inhibitor of WNT Production-2	Merck	Cat# 10536
Smoothened agonist	Merck	Cat# 566660
Matrigel	Corning	Cat# 354234
Triiodothyronine	Sigma	Cat# T2877
Biotin	Sigma	Cat# B4639
Recombinant human neurotrophin-3	PeproTech	Cat# 450–03
Recombinant human/murine/rat brain-derived neurotrophic factor	PeproTech	Cat# 450–02
Cyclic adenosine monophosphate	Sigma	Cat# D0627
Recombinant human hepatocyte growth factor	PeproTech	Cat# 315–39
Recombinant human insulin-like growth factor 1	PeproTech	Cat# 100–11
Recombinant human platelet-derived growth factor AA	R&D Systems	Cat# 221-AA
B27® supplement (50X)	Gibco	Cat# 17504–044
2-Phospho-L-ascorbic acid trisodium salt	Sigma	Cat# 49752
HEPES buffer solution (1M)	Gibco	Cat# 15630–056
Hanks' Balanced Salt Solution without Ca^2+^ and Mg^2+^	Thermo Fisher	Cat# 14025092
Low-melt agarose	Sigma	Cat# A9414-100G
StemPro® Accutase®	Gibco	Cat# A11105-01
Geltrex™	Gibco	Cat# A15696-01
37% Formaldehyde solution	Carl Roth	Cat# CP10.1
Sucrose	Sigma	Cat# S9378
Gelatin	NeoLab	Cat# 9475.0500
Triton X-100	Fisher Scientific	Cat# BP151-100
Bovine serum albumin	Carl Roth	Cat# 8076.3
Fluorescence mounting medium	Dako	Cat# S3023
CUBIC-L	TCI	Cat# T3740
CUBIC-R(M)	TCI	Cat# T3741
QIAzol Lysis Reagent	Qiagen	Cat# 79306
Hot FIREPol EvaGreen qPCR Mix Plus	Solis Biodyne	Cat# 08-24-0000S
NB + medium	ThermoFisher Scientific	Cat# 21103049
glutamine	ThermoFisher Scientific	Cat# A2916801
B-27+ supplement	ThermoFisher Scientific	Cat# 17504044
Calbryte™ 520 a.m.	AAT Bioquest, Inc.	Cat# 20650

**Critical commercial assays**		

Amaxa™ P3 Primary Cell 4D Nucleofector X Kit	Lonza	Cat# V4XP-3024
24XCyte mFISH probe kit	Metasystems	Cat# D-0125-120-DI
RNeasy Mini Kit	Qiagen	Cat# 74106
RNase-free DNase Set	Qiagen	Cat# 79254
RevertAid RT Kit	Life Technologies	Cat# K1691
10X Genomics® Chromium™3′gene expression RNA-seq library preparation (by Azenta)	10X Genomics	NA
10X Genomics® library sequencing on the Illumina series (by Azenta)	10X Genomics	NA
CyQUANT™ LDH Cytotoxicity Assay Kit	Invitrogen	Cat# C20301

**Deposited data**		

human reference transcriptome (GRCh38) for gene mapping using Cell Ranger pipeline (version 7.0.1 (10X Genomics)	10X Genomics	refdata-gex-GRCh38-2020-A
RNA-seq	Gene Expression Omnibus (GEO)	GEO: GSE301276
snRNA-seq	Gene Expression Omnibus (GEO)	GEO: GSE301277
Raw data depository	Zenodo	Zenodo: https://doi.org/10.5281/zenodo.15525634

**Experimental models: Cell lines**		

Human: ES cell line WA09-FI (H9)	WiCell Research Institute, Wisconsin, USA	LOT# WB66595
Human: glioblastoma cell line U87 (HTB-14)	ATCC	LOT# 63710285

**Oligonucleotides**		

*18S rRNA*	biomers.net GmbH	NR_003286.2
*c-MYC*	biomers.net GmbH	NM_002467.6/NM_001354870.1
*TP53*	biomers.net GmbH	NM_001407266.1
*MKI67*	biomers.net GmbH	NM_002417.5
*NF1*	biomers.net GmbH	NM_000267.3
*PTEN*	biomers.net GmbH	NM_001304718.2
*PROM1*	biomers.net GmbH	XM_054351160.1
*GLS*	biomers.net GmbH	XM_054341407.1
*SNAI1*	biomers.net GmbH	NM_005985.4
*MAP2*	biomers.net GmbH	NM_002374.3
*GFAP*	biomers.net GmbH	NM_002055.5
*VIM*	biomers.net GmbH	NM_003380.5
*MBP*	biomers.net GmbH	NM_001025081.1
*DCX*	biomers.net GmbH	NM_178152.3

**Recombinant DNA**		

*SB100X* vector	VectorBuilder	Cat# VB190927-1101xtm
*MYC* with *EGFP* vector	VectorBuilder	Cat# VB190927-1100geg

**Software and algorithms**		

Excel (2016)	Microsoft Corporation	N/A
Metafer5 software	MetaSystems Hard & Software GmbH	N/A
VSlide Viewer	MetaSystems Hard & Software GmbH	N/A
LAS X software (v3.5.7.23225)	Leica Microsystems GmbH	N/A
ImageJ (v1.53i)	National Institute of Health (NIH)	N/A
Imaris Viewer	Oxford Instruments	N/A
QuantStudio™ Design & Analysis software (v1.5.1)	Thermo Scientific	N/A
FastP preprocessor	GitHub	https://github.com/OpenGene/fastp
STAR Aligner	Cold Spring Harbor Laboratory	http://code.google.com/p/rna-star/
Bioconductor	Bioconductor	https://www.bioconductor.org/
Huygens™ Essential	Scientific Volume Imaging	N/A
Loupe browser	10X Genomics	N/A
R Studio (v 4.5.0)	Posit Software, PBC	https://www.r-project.org/
Seurat package 5.2.0 and Seurat workflow	Satija Lab and Collaborators	https://satijalab.org/seurat/
GraphPad Prism (v 9.3.1)	GraphPad Software	N/A

**Other**		

Human adult brain total RNA	Clontech	Cat# 636530
Human fetal brain total RNA	Clontech	Cat# 636526

### Experimental model and study participant details

#### Culture of human embryonic stem cell line WA09-FI (H9)

The feeder-independent human embryonic stem cell line WA09-FI (H9) was used for all studies presented as approved according to §4 and §6 of the German Stem Cell Act (registry numbers 3.04.02/0125 and 3.04.02/0125-E01). The line was originally generated by the group of Dr. James Thomson at the University of Wisconsin^97^ and contains an XX karyotype. H9 cells (LOT: WB66595) were obtained from the WiCell Research Institute, Wisconsin, USA, at passage 23 and were used for experiments in passages 34–56. Authentication was performed by the provider by short tandem repeat analysis and karyotype analysis (G-banding). The authors performed addition mFISH analyses and immunocytochemical analyses for embryonic stem cell markers. H9 cells were routinely cultured on Laminin-521-coated culture dishes (BioLamina, #600962, 10 μg/mL) in mTeSR1 basal medium medium with addition of 1x mTeSR1 supplement (STEMCELL Technologies, #85851 and #85852) and 50 U/ml penicillin and 50 μg/mL streptomycin (Merck, #A2212) or in TeSR™-E8™ medium with 1xTesr™-E8™ supplement (STEMCELL Technologies, #05991 and #05992). Cells were incubated at 37°C, 5% CO_2_, atmospheric O_2_ content, and relative humidity of 95% and passaged every week using ReleSR (STEMCELL Technologies, #05872). Briefly, medium was aspirated, and cells were rinsed first with 2 mL PBS without calcium chloride and magnesium chloride (PBS−/−) and then with 200 μL ReleSR. After aspiration of the non-enzymatic passaging reagent, cells were incubated for 2 min (min) at 37°C to detach only pluripotent cells. Detachment was stopped by addition of pre-warmed medium, and cells were seeded to about 1.0 x10^5^ cells per 60 mm Petri dish.

#### Culture of U87 cell line

The human glioblastoma cell line U87 (karyotype XY) was purchased from ATCC® (HTB-14, LOT: 63710285) and used in passage 128 to 140. Authentication was performed by the provider. U87 cells were used for comparison with GFP^+^/c-MYC^high^ cells. Cells were routinely cultured in T25 flasks in DMEM+GlutaMAX™-1 (Gibco, #31966-021) with 10% fetal bovine serum superior (Sigma-Aldrich, #S0615), 100 U/ml penicillin, and 100 μg/mL streptomycin (Merck, #A2212). Incubation was performed at 37°C, 5% CO_2_, atmospheric O_2_ content, and relative humidity of 95% and cells were passaged once a week. For this purpose, medium was aspirated, cells were rinsed with PBS−/−, and evenly covered with 1 mL Trypsin and incubated for 5 min at 37°C. Detachment was stopped by addition of pre-warmed medium, cell suspensions of two to three T25 flasks were collected in one 15 mL tube, and centrifugation at 300 xg for 5 min. Afterward, the supernatant was discarded, cells were resuspended in medium, and seeded to about 1.0 x10^5^ cells per T25 flask.

Due to the nature of the tumor model based on cMYC overexpression, we do not expect sex/gender-associated differences. All cell lines were regularly tested for mycoplasma contamination via PCR analysis.

### Method details

#### Generation of cerebral organoids

Cerebral organoids were generated as previously described^23^^,^^24^ and incubated at 37°C, 5% CO_2_, atmospheric O_2_ content, and relative humidity of 95%. Briefly, for the generation of embryonic bodies (EBs), H9 cells were detached using ReLeSR (Stemcell Technologies, #05872) for 3 min at 37°C. 18000 cells in TeSR™-E8™ medium with addition of 50 μM Rho-associated protein kinase (ROCK) inhibitor (Tocris Bioscience, #1254), to enhance the survival of the dissociated cells by preventing apoptosis and to improve EB formation, were plated into each well of an U-bottom suspension plate (Sarstedt) pre-coated with anti-adherence solution (Stemcell Technologies, #07010) for 5 min, 1000 rpm, at RT. Five to ten 0.5 to 1 mm long microfilaments (surgical suture; Johnson & Johnson International, #V632H) or 0.4% polyvinyl alcohol (PVA; Sigma-Aldrich, #V632H) were used to enhance the assembly of the embryoid body to give rise to the organoid.^98^^,^^99^ Medium was changed to Essential 6™ Medium (Gibco, # A1516401) supplemented with two SMAD pathway inhibitors named dorsomorphin (2.5 μM, Biogems, #8666430) and SB-431542 (10 μM, Torcris, #1614) two days after seeding. This day is defined as the start of differentiation and therefore, the age of the organoids was calculated from this day (0 days old; d0). For the first 5 days, Essential 6™ medium was changed every day and supplemented with dorsomorphin and SB-431542 to derive neural progenitor cells from hES cells. On day 6, the organoids were transferred to 24-well suspension plates and cultured in an improved differentiation medium – A (IDM-A, according to Lancaster et al., 2017) containing 1:1 Dulbecco’s Modified Eagle Medium/Nutrient Mixture F-12 (DMEM/F12; Gibco, #31330-038) and Neurobasal® (Gibco, #21103-049), 0.5% *N*-2 supplement, 2% B27® – vitamin A (Gibco, #12587-010), 0.25% insulin (Sigma, #19278), 50 μM β-mercaptoethanol (Sigma, #M6250), 1% GlutaMAX™-1 (Gibco, #35050-038), 0.5% Minimum Essential Medium – Non-essential amino acid solution (MEM-NEAA; Gibco, #11140-035), and 1% penicillin-streptomycin (Merck, #A2212). The IDM-A was supplemented with 20 ng/mL epidermal growth factor (EGF, Peprotech, #AF-100-15) and 20 ng/mL basic fibroblast growth factor (bFGF, Peprotech, # 100-18b) for 19 days (until day 24) with daily medium change in the first 10 days, and every other day for the subsequent 9 days, to promote neural and glial lineage differentiation. Additionally, 5 μM of the inhibitor of WNT Production-2 (IWP-2, Merck, #10536) was added to the medium from day 4 until day 24 to inhibit the WNT pathway, and 1 μM of the small molecule smoothened agonist (SAG, Merck, #566660) was added from day 12 to day 24 to activate the sonic hedgehog (SHH) pathway, to promote further neuronal proliferation and maturation. EBs were embedded into Matrigel (Corning, #354234) droplets by incubation of each EB within 30 μL air bubble free Matrigel for 27 min at 37°C on day 15 and cultured in IDM-A with addition of EGF/bFGF, IWP2 and SAG with medium being exchanged every second day. From day 25–36, organoids were cultured in IDM-A supplemented with 60 ng/mL Triiodothyronine (T3, Sigma, #T2877), 100 μg/mL biotin (Sigma, #A4639), 20 ng/mL neurotrophin-3 (NT-3, Preprotech, #450-03), 20 ng/mL brain-derived neurotrophic factor (BDNF, Peprotech, #450-02), 10 μM cyclic adenosine monophosphate (cAMP, Sigma, #D0627), 5 ng/mL hepatocyte growth factor (HGF, Peprotech, #315-39), 10 ng/mL insulin-like growth factor 1 (IGF-1, Peprotech, #100-11), and 10 ng/mL platelet-derived growth factor AA (PDGF-AA, R&D Systems, #221-AA), to promote OPC survival and proliferation. To allow homogeneous distribution of the different factors and nutrients, on day 25, the organoids were transferred to T25 suspension flasks with filtered cap and put on an orbital shaker with a throw of 19 mm and a corresponding speed of 62 r.p.m. From day 37 onwards, organoids were cultured in IDM+A, namely IDM-A containing 2% B27® + vitamin A (Gibco, #17504-44), plus 0.4 mM vitamin C (Sigma, #49752) and 12.5 mM HEPES Buffer Solution (Gibco, #15630-056) to control pH levels. The medium was again supplemented with T3, biotin and cAMP to promote oligodendrocyte maturation. Media changes were performed every three days until day 49, then every three to four days for the duration of the culture.

#### Air-liquid interface culture

Culture of organoids at air-liquid interface was performed according to the protocol by Giandomenco and colleagues^26^ with slight modifications. Briefly, 3–6 cerebral organoids between 50 and 60 days old were collected and washed in Hanks' Balanced Salt Solution without Ca^2+^ and Mg^2+^ (HBSS, Thermo Fisher, #14025092) and embedded in 3% low-melt agarose (Sigma, # A9414-100G) at approximately 37°C in peel-a-way® embedding molds (Sigma). The agarose blocks with embedded organoids were incubated on ice for 10–15 min and cut into 300 μm thick sections in cold HBSS using Leica VT1200S vibratome. An amplitude of 1 mm, a speed of 0.30 mm/s, and a razor blade angle of 21° were used. Sections were collected onto Sarstedt TC-inserts (pore size of 0.4 μm) in 12-well plates and first left to equilibrate for 1 h at 37 C in IDM+A and addition of 100 μg/mL Biotin, 60 ng/mL T3 and 10 μM cAMP. Cultured slices were maintained at the air-liquid interface in IDM+A medium with the addition of mentioned supplements at 37°C, 5% CO_2_, atmospheric O_2_ content, and relative humidity of 95% with daily medium changes (3/4 of the medium).

#### Genetic modifications in organoids

Overexpression of c-*MYC* oncogene in cells of organoids was based on the *Sleeping Beauty* transposon system consisting of two vectors: *SB100X* (VB190927-1101xtm) and c-*MYC* (VB190927-1100geg) expressing vector (VectorBuilder; Figure S6).

Cerebral organoids were subjected to nucleofection on day 11 of the culture using Amaxa™ P3 Primary Cell 4D Nucleofector X Kit (Lonza, #V4XP-3024) according to the manufacturer’s protocol. Briefly, 8–15 organoids were placed between electrodes of the cuvette and the excess medium was removed. Then, nucleofection medium consisting of premixed solution (82 μL) and supplement (18 μL) from the kit with addition of plasmid vectors, *SB100X* (2 μg) and *c-MYC* (1.1 μg or 2.2 μg) expressing vector (VectorBuilder), was added to organoids. Sham-nucleofected organoids served as a negative control. Nucleofection program CB-150 for H9 cells was applied and organoids were immediately placed at 37°C and 5% CO_2_ for 10 min, following addition of culture medium to the cuvette and incubation for the next 10 min. Then, organoids were placed in a suspension Petri dish, maintained in IDM-A medium with addition of EGF, bFGF, and IWP-2 and incubated at 37°C, 5% CO_2_, atmospheric O_2_ content, and relative humidity of 95%. Medium was exchanged according to the same protocol as described for normal control organoids in a previous section. Nucleofected organoids were cultured in parallel with control sister organoids (i.e., from the same batch). Organoids were monitored daily for the presence of GFP signal that was associated with successful nucleofection.

#### Isolating GFP^+^/c-MYC^high^ cells from cerebral organoids

Nucleofected organoids (2–8 per sample) showing large areas with GFP signal were selected for isolation of genetically modified cells at day 50–91 of the culture. Organoids were then washed in PBS−/− and dissociated by mincing and using StemPro® Accutase® (Gibco, #A11105-01) in a 15–30 min incubation in a microtube at 37°C and 5% CO_2_ on an orbital shaker. Additionally, every 10 min the suspension was pipetted up and down and the supernatant containing single dissociated cells was transferred to a new microtube and centrifuged at 300 xg for 5 min or 200 xg for 7 min at RT. The cell pellet was resuspended in PBS−/− with addition of 50 μM Rock inhibitor (Tocris Bioscience, #1254) and placed on ice to minimize the cell death. The remaining non-dissociated organoid pieces were used to gain more cells by repeating the incubation with new addition of StemPro® Accutase®, centrifugation at 300 xg for 5 min or 200 xg for 7 min at RT. Cell suspension from the same condition was merged after dissociation and filtered using a cell strainer with 35 μm nylon mesh (Corning) into a cell sorting tube. Cells were immediately sorted by fluorescence-activated cell sorting (FACS) based on the presence of GFP signal. Sham-nucleofected or control organoids dissociated using the same procedure were used as a negative control for adjustment of gates for cell populations prior to sorting. Positively selected (GFP^+^) cells were either used directly for ALI-culture or plated onto Geltrex™(Gibco, #A15696-01)-coated petri dishes and cultivated in IDM+A supplied with 60 ng/mL T3, 100 μg/mL biotin, and 10 μM cAMP at 37°C, 5% CO_2_, atmospheric O_2_ content, and relative humidity of 95% for their propagation and used for generation of tumor spheres.

#### Generation of the tumor models

Isolated GFP^+^/c-MYC^high^ cells were used to generate tumor spheres. For this, cells were detached by washing with PBS−/− and incubation for 7 min at 37°C within StemPro® Accutase®. Afterward, cells were transferred with DM + A into a 1.5 mL low binding microtube, centrifuged at 200 xg for 7 min, and the supernatant was discarded. The cells were resuspended in Matrigel on ice (1x10^4^ cells mixed with 5 μL Matrigel (Corning, #354234) and incubated for 10 min at 37°C for polymerization. Tumor spheres were cultivated in IDM+A supplied with 60 ng/mL T3, 100 μg/mL biotin, and 10 μM cAMP at 37°C, 5% CO_2_, atmospheric O_2_ content, and relative humidity of 95%. One day after tumor sphere generation, assembloid were generated by cultivation of one organoid and one tumor sphere together in one well of a 24-well suspension plate at an angle on an orbital shaker with 19 mm throw at 114 r.p.m at 37°C, 5% CO_2_, atmospheric O_2_ content, and relative humidity of 95%. The exchange of IDM+A was performed every two days, while the medium was supplied with T3, biotin and cAMP. Alternatively, GFP^+^ cells were applied directly after sorting on top of the slice at ALI-culture (1x10^3^ cells/organoid slice) and incubated at 37°C, 5% CO_2_, atmospheric O_2_ content, and relative humidity of 95%.

#### Chromosome preparation and M-FISH

On Petri dishes cultured cells were incubated with 10 μL/mL colcemide at 37°C, 5% CO_2_, atmospheric O_2_ content, and relative humidity of 95% (2.5 h for H9 and 4 h for GFP^+^/c-MYC^high^ cells) and harvest as described above. After centrifugation at 200 xg for 6 min, supernatant was removed, cells were resuspended drop by drop with 37°C 0.075 M KCl solution, and incubated at RT (12 min for H9 and 40 min for GFP^+^/c-MYC^high^ cells). Afterward, cells were fixed with 3:1 methanol/glacial actic acid for 30 min at RT and cell suspension was dropped on wet slides. After drying, chromosomes were hybridized with the 24XCyte mFISH probe kit (Metasystems, D-0125-120-DI) according to the protocol recommended by the manufacturer and counterstained with DAPI/antifade-solution (Metasystems). Metaphase images were captured with the fluorescence microscope Zeiss Axio Imager.Z2 equipped with Metafer5 software (Metasystems) and analyzed using Isis/mFISH software (Metasystems).

#### Immunofluorescence staining (IF)

Organoids and organoid slices were fixed in 3.7% formaldehyde (Carl Roth, #CP10.1) at 4°C overnight and then washed three times for 5 min with PBS. Organoids were dehydrated in sucrose gradient (7%, 10% 30%, 40% 60% sucrose in PBS; 4h for each step except overnight for 30% and 60% sucrose at 4°C). Organoid slices were dehydrated in 30% sucrose (Sigma, #S9378) in PBS overnight. Organoids were then embedded in 7.5% gelatin (Neolab, #9475.0500)/10% sucrose using in house 3D-printed PDMS embedding molds. Embedded organoids were frozen on dry ice and stored at −80°C prior cryosectioning at 10 μm (if not stated differently) using CM1860 cryostat (Leica Biosystems). For staining against MBP, antigen retrieval was performed using Tris-EDTA (pH 9) for 30 min in water bath at 95°C. Cryosections were blocked and permeabilized in 0.5% Triton X-100 (ThermoFisher Scientific, #BP454-100)/1% BSA (Carl Roth; #8076.3) in PBS for 30 min and blocked with 1% BSA in PBS for 30 min at room temperature (RT). Incubation of samples with primary antibodies in 1% BSA in PBS was performed either for 1 h at RT or at 4°C overnight following washing three times for 5 min with PBS. If the primary antibody was not directly conjugated to a fluorescent dye, samples were then incubated with secondary antibodies in 1% BSA in PBS for 1h at RT followed by washing three times for 5 min with PBS and incubation with 5 μg/mL DAPI for 4 min for nuclei staining. Then, the samples were washed two times for 5 min in PBS followed by washing for 5 min with Millipore water prior to mounting with fluorescence mounting medium (Dako, #S3023). The primary and the secondary antibodies used for immunofluorescence are listed in Tables S1–S3. Images were captured with a fluorescence microscope Zeiss Axio Imager.Z2 equipped with Metafer5 software (Metasystems) and with a confocal microscope DMI 4000B (Leica Microsytems) with LAS X software (v3.5.7.23225, Leica Microsystems). The stainings were performed on samples from three independent preparations with at least two organoids per group. Images were processed with VSlide Viewer (Metasystems) and ImageJ (v1.53i, National Institute of Health (NIH)), with brightness and contrast adjusted for better visibility. By compiling z-stacks, 3D images were generated using Imaris Viewer (Oxford Instruments).

#### Clearing and IF for light sheet microscopy

Clearing, staining and imaging of whole organoids were performed according to the modifications of the CUBIC protocol.^100^^,^^101^^,^^102^ Fixed, whole organoids were treated with 50% CUBIC-L (TCI, #T3740) in Millipore overnight at 37°C on a shaker set to 144 rpm. The organoids were then incubated in CUBIC-L at 37°C for 2–3 days with agitation at the same speed, followed by three washing steps with PBS, 1 h each at RT. Subsequent incubations were performed at RT with gentle agitation (60 rpm), taking care to avoid damage of organoids during solution changes. For immunofluorescence staining, the organoids were blocked with 5% BSA in PBS for 3 h at 37°C and incubated with primary antibodies diluted in 5% BSA in PBS for 24 h. The following day, organoids were washed three times with PBS (1 h each) and incubated with the corresponding secondary antibodies in 5% BSA in PBS for 24 h. Afterward, unbound antibodies were removed with three 1-h washes in PBS. If applicable, conjugated antibodies diluted in 5% BSA in PBS, were applied and incubated for 24 h. For the final step, the last antibody solution was washed out with three 1-h PBS washes. The organoids were then stained with DAPI (5 μg/mL) for at least 12 h and were washed three times with PBS (1 h each) afterward. To match the refractive index, the organoids were first treated with 50% CUBIC-R(M) (TCI, #T3741) in Millipore for 12 h, followed by incubation in CUBIC-R for 2 days, with the solution refreshed after 24 h. Cleared and stained organoids were imaged using a Leica STELLARIS DLS system with an HC APO L 10x/0.30 W DLS objective and a 7.8 mm TwinFlect mirror cap. Samples were mounted in a 35 mm glass-bottom dish (ibidi), with a base layer of 4% agarose/CUBIC-R poured and set for 30 min. The organoids were then embedded on top of this layer in the same matrix. Areas for the mirror cap were cut out, and the dish was filled with CUBIC-R solution before immersion of the objective. Images were processed with ImageJ (v1.53i, National Institute of Health (NIH)) and Huygens™ Essential (Scientific Volume Imaging).

#### Real time RT-PCR analysis

C-MYC^high^ cells, tumor spheres (n = 4–6 per batch), whole organoids (*n* = 3 per group) or organoid slices (*n* = 3 per group) were collected separately or pooled in QIAzol Lysis Reagent (Qiagen, #79306) and total RNA was isolated using the Qiagen RNeasy Mini Kit (#74106) according to the manufacturer’s instructions including a DNA removal step using the RNase-free DNase Set (Qiagen, #79254). 50 ng RNA were reverse-transcribed via the RevertAid RT Kit (Life Technologies, #K1691) and the PCR Thermal Cycler PeQSTAR (PeQLab), following the program 5 min 25°C, 60 min 42°C, and 5 min 70°C. Relative RNA expression of each gene was analyzed in technical triplicates using the Hot FIREPol EvaGreen qPCR Mix Plus from Solis Biodyne (#08-24-0000S) and the QuantStudio 3 Real-Time PCR System (Thermo Scientific), following the program 15 min 95°C for initial denaturation, 45 cycles of 15 s 95°C, 20 s 60°C, and 20 s 72°C for PCR, and 1 cycle of 15 s 95°C, 60 s 60°C, and 1 s 95°C for melt curve analysis. The standard curve for each primer was generated using either human fetal and or adult brain mRNA (2 μg–3.2 ng). Data were analyzed using QuantStudio™ Design & Analysis software (v1.5.1, Thermo Scientific) and Excel (2016, Microsoft Corporation). Target expression level of each gene was normalized to *18S rRNA*. Primer sequences are listed in Table S4.

#### Lactate dehydrogenase (LDH) level

Extracellular lactate dehydrogenase (LDH) released from necrotic cells in the media was determined using CyQUANT™ LDH Cytotoxicity Assay Kit (Invitrogen, #C20301) according to manufacturer’s instructions. Briefly, culture medium was collected at approximately day 100 of the culture and stored at −80°C. On the day of assay, 50 μL of each sample medium was thawed on ice, added to a 96-well black clear-bottom plate in duplicate wells. Then, 50 μL of Reaction Mixture from the kit was added to each sample and incubated at RT for 30 min in the dark to enable conversion of lactate to pyruvate in a reaction catalyzed by LDH where NAD+ is reduced to NADH. The reaction was stopped by adding 50 μL Stop Solution from the kit. The absorbance of red formazan product generated by reduction of tetrazolium by diaphorase, which also oxidizes NADH, was measured spectrophotometrically at 490 nm. Using Excel (2016, Microsoft Corporation), the 680-nm absorbance value (background) was subtracted from the 490-nm absorbance. Obtained absorbance values are directly proportional to the amount of LDH released into the media.

#### Ca-imaging

The samples were cultured in the air-liquid interface as previously described. To avoid damaging the outgrowths of the samples, the slices were left on the air-liquid interface. One day before staining, the slices were covered with NB + medium (NB + medium (ThermoFisher Scientific, 21103049), 0.5 mM glutamine (ThermoFisher Scientific, A2916801), 2% B-27+ supplement (ThermoFisher Scientific, 17504044) and 1% Pen/Strep. The next day, the slices were loaded with a solution of 5 μM Calbryte 520 AM (AAT Bioquest, Inc., 20650) in NB + medium and incubated at 37°C for 60 min. The dye solution was replaced with fresh medium to remove excess probes. Prior to recording, samples were stored in the incubation chamber of the microscope at ∼37°C for an additional 15 min. The recordings were performed with a confocal inverted microscope, Leica DMI8-CS, Stellaris 5, and a multi-immersion objective HC PL APO 20x/0.75 IMM CORR CS2. The tunable Wightlight laser (WLL) was set to a wavelength of 493 nm. A maximum laser power of 15 per cent was used to protect the samples. The video sequences were recorded at 4 Hz and a size of 512 x 512 pixels. Multiple cells were segmented and analysed using the open source software ImageJ (FIJI).^103^ The fluorescence change over time was defined as ΔF/F = (F0 −F1)/(F0), where F0 represented the 10th percentile of the signal in a rolling window.

#### Scratch assay

Isolated GFP^+^ cells and U87 cells were seeded one day in advance on Geltrex™(Gibco, #A15696-01)-coated 6-Well plates depending on the proliferation rate in a density of 0.15x10^6^ to 0.5x10^6^ cells per well and incubated at 37°C, 5% CO_2_, atmospheric O_2_ content, and relative humidity of 95%. The cell layers were manually scratched using a 200 μL pipette tip, washed with the respective medium, and incubated in 4 mL of the respective medium at 37°C, 5% CO_2_, atmospheric O_2_ content, and relative humidity of 95% afterward. Scratches were documented using the Revolve R4 microscope from ECHO (10× objective from Olympus) immediately after scratching and afterward every 24 h.

#### Bulk RNA sequencing

Organoid slices (*n* = 3), organoids (*n* = 3), assembloids (*n* = 3), tumor spheres (*n* = 6), each pooled from the same biological replicate in one tube, and tumor-like cells (*n* = 1), were washed in cold PBS at 300xg, 5 min. RNA was isolated as described in a section “real time RT-PCR analysis” and stored at –80°C. Preparation of samples for sequencing including the isolation of the nuclei, initial quality control, library preparation, sequencing and initial data processing were performed as a service by GENEWIZ/AZENTA (USA). The standard RNA-seq bundle with polyA selection with 2x150 bp was performed on the Illumina series. The estimated sequencing output was ∼50 million paired-end reads per sample. Raw reads were preprocessed using FastP preprocessor (https://github.com/OpenGene/fastp) for trimming adapters, polyG or polyX, and aligned to the human genome (hg38) using the STAR Aligner (http://code.google.com/p/rna-star/). Data analysis of the filtered feature counts was performed using the R software (https://www.r-project.org/) with Bioconductor (https://www.bioconductor.org/).

#### Single nuclei RNA sequencing

Organoid slices (*n* = 3) and organoids (*n* = 3), each pooled from the same biological replicate in one tube, were washed in cold PBS at 300xg, 5 min. After complete removal of PBS, organoid slices and organoids were placed on dry ice for 5 min, and then stored in liquid nitrogen. Preparation of samples for sequencing including the isolation of the nuclei, initial quality control, library preparation, sequencing and initial data processing were performed as a service by GENEWIZ/AZENTA (USA). The RNA-seq library was prepared using 10X Genomics Chromium® 3` expression, and the 10X library sequencing was performed on the Illumina series. The target number of cells per sample was 6000, and number of reads per cell 50000. Raw reads were preprocessed and aligned to the human genome (hg38) using the Cell ranger pipeline (10X Genomics). Initial quality control and visualization of the data was performed in the Loupe browser (10X Genomics). Data analysis of the filtered feature matrix was performed using the R software (https://www.r-project.org/) with Seurat package and Seurat workflow (https://satijalab.org/seurat/). Cells that had unique feature counts over 2500 or less than 200, and cells that had >5% mitochondrial count were filtered out from the dataset. The data was normalized using a SCTransform function for data normalization, scaling and reduction prior to graph-based clustering and visualization of the merged and integrated datasets for organoids and organoid slices.

#### Irradiation with X-Rays

Whole organoids at day 100 of the culture, whole organoids at day 100 of the culture assembled with tumor spheres, and tumor spheres were irradiated using the X-ray tube MXR-320/26 from Comet with 250 kV, 16 mA, and a dose rate of 1.3 Gy/min for organoids and 3 Gy/min for organoid slices. The medium was exchanged immediately after irradiation. Irradiated samples were compared to sham-irradiated controls (0 Gy).

### Quantification and statistical analysis

All analyses were performed for independent experiments (N) containing multiple organoids (n) and using GraphPad Prism (v 9.3.1). Normal distribution of studied variables was proved by normality and lognormality tests with accompanied QQ-plots, and homogeneity of variances was checked using F-test. Statistical comparisons were performed as stated in figure legends and included unpaired two-tailed test with or without Welch correction, Brown-Forsythe/Welch, or mixed ANOVA model either with Dunnett′s or Tukey’s post-test. ∗*p* < 0.05, ∗∗*p* < 0.01, ∗∗∗*p* < 0.001, ∗∗∗∗*p* < 0.0001. Samples of organoid slices were randomly assigned to different conditions or treatments. No statistical methods were used to pre-determine sample sizes. Because of the nature of the treatment (nucleofection), data collection and analysis were not performed blind to the conditions of the experiments.
