# Supplementary material for: Human cerebral organoids model tumor initiation and infiltration in an autologous astrocyte-supported setting
Source: iScience. 2025 Aug 11;28(9):113334. doi: 10.1016/j.isci.2025.113334 (PMC12410359; doi:10.1016/j.isci.2025.113334)
Supplement: Document S1. Figures S1–S8 and Tables S1–S4 [file mmc1.pdf]

iScience, Volume *28*

Supplemental information

Human cerebral organoids model tumor initiation and infiltration in an autologous astrocyte-supported setting

Esther Schickel, Tamara Bender, Leon Kaysan, Simone Hufgard, Margot Mayer, David R. Grosshans, Christiane Thielemann, and Insa S. Schroeder

Supplementary items and legends

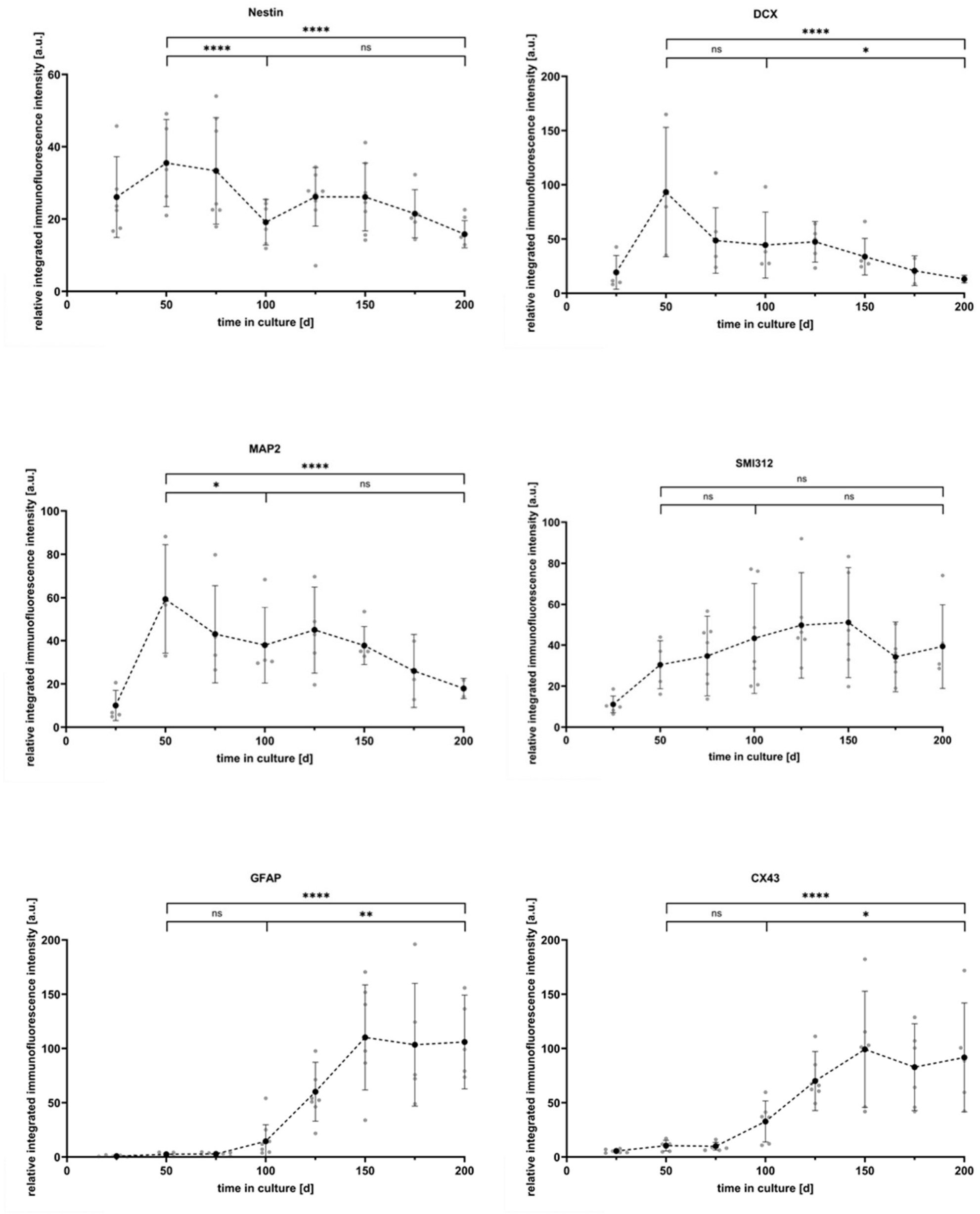

Supplementary Figure 1. Maturation of cerebral organoids, Related to Figure 2. Relative immunofluorescence intensity of nestin, DCX, MAP2, SMI312, GFAP, and CX43 normalized to the related nuclei area. For each marker, four whole organoid sections from organoids at day 25, 50, 75, 100, 125, 150, 175, and 200 of the culture were analyzed. Data are presented as mean ± SD for two to three independent experiments (N = 2-3) and one to three organoids per experiment (n = 1-3), * $p<0.05$, ** $p<0.01$, *** $p<0.001$, **** $p<0.0001$. Statistical analysis was done using one-way ANOVA with Tukey's post-test (nestin, MAP2, and SMI312) or with Kruskal-Wallis Test with Dunn's post-test.

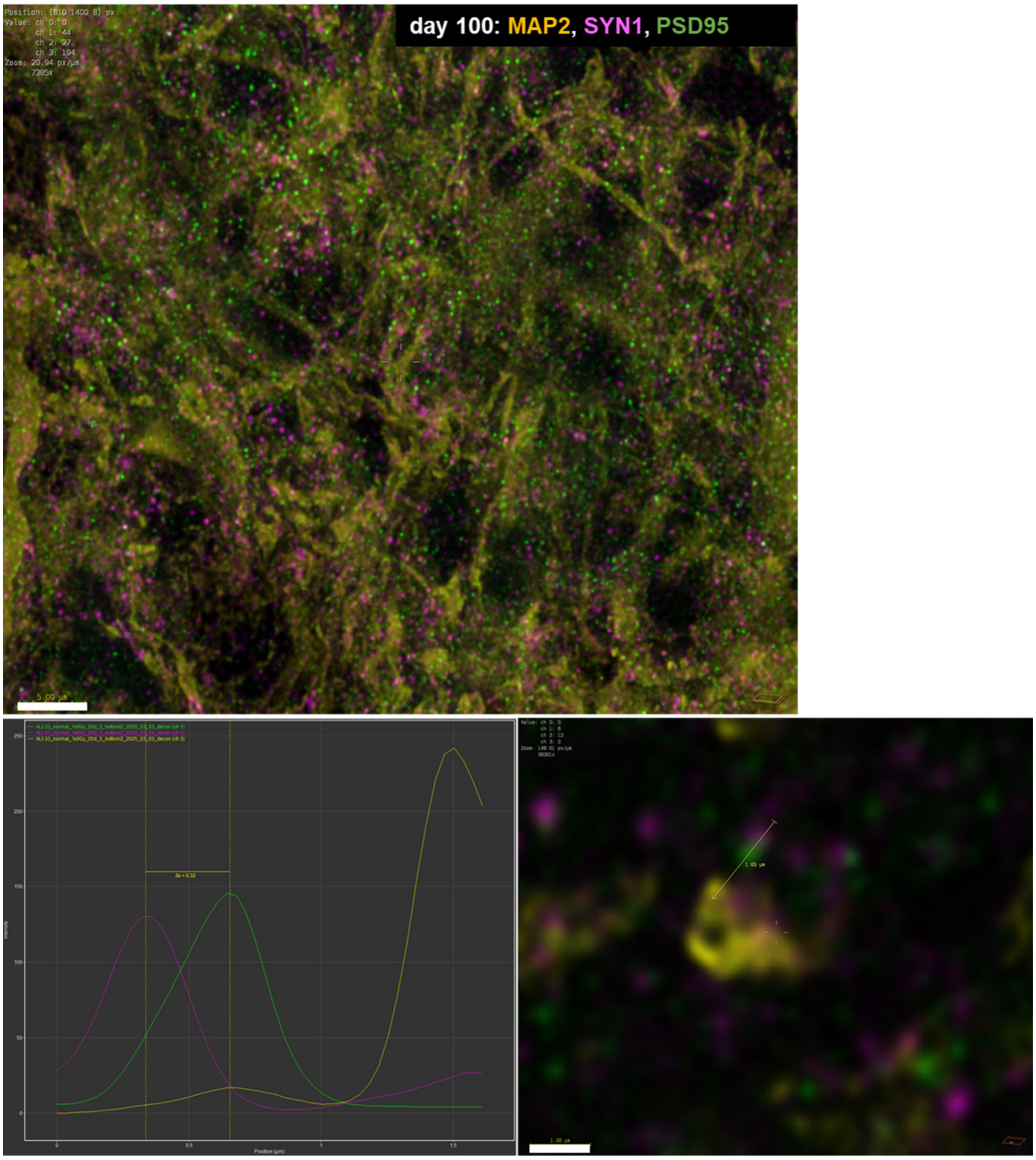

Supplementary Figure 2. Visualization of the synaptic cleft in organoids at day 100 of the culture, Related to Figure 2. Intensity measurement of fluorescent signals for presynapse (SYN1, magenta) and postsynapse (PSD95, green) (left image). The x-axis is defined by the arrow shown in the right picture. Even with confocal imaging, the small synaptic cleft (200 to 300 nm) can be measured/visualized, especially after deconvolution. The pictures show the data after deconvolution. Scale bar: 5 µm (upper picture) 1 µm (lower right picture).

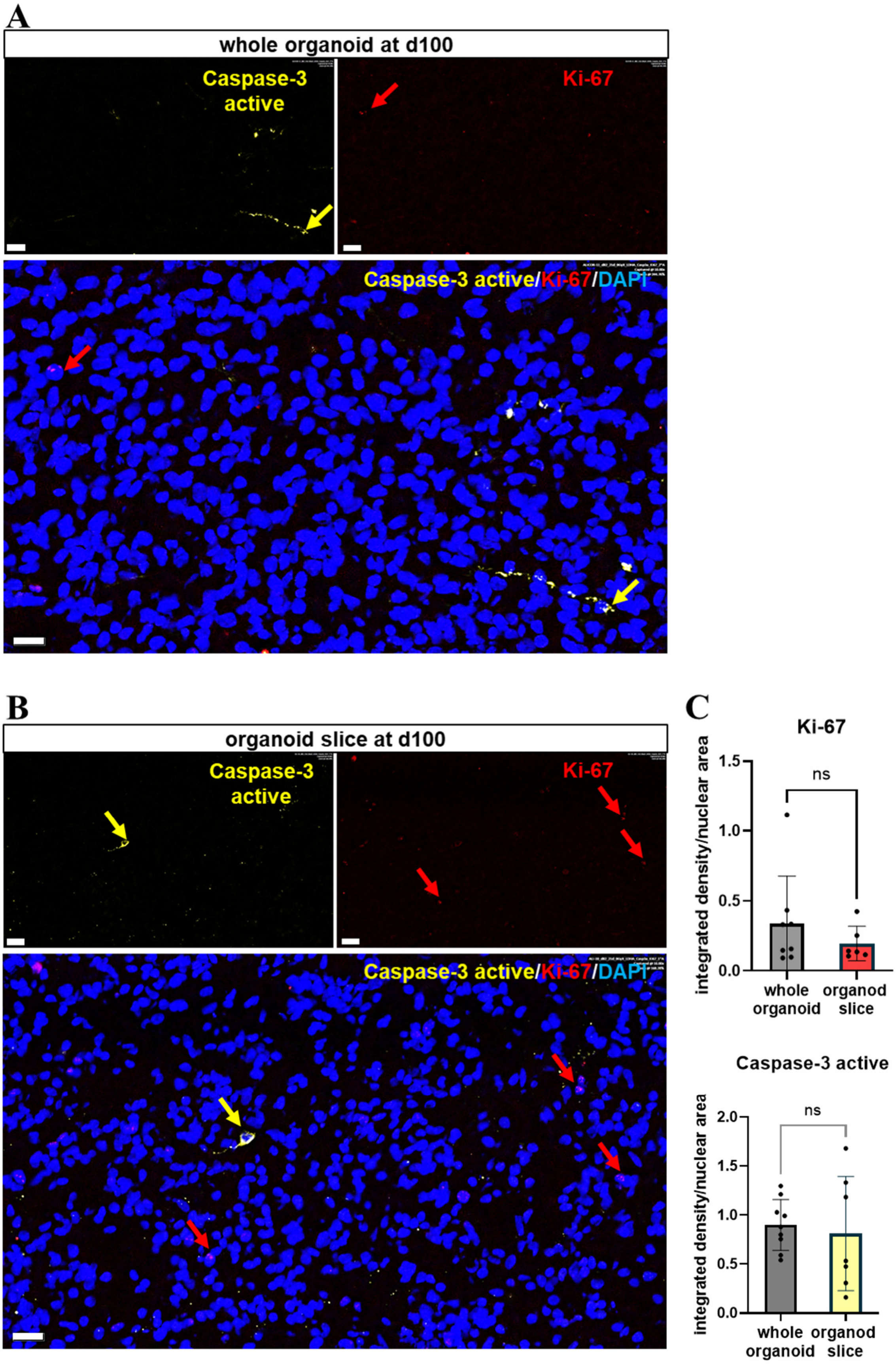

Supplementary Figure 3. Proliferation and apoptosis in whole organoids and organoid slices, Related to Figure 3. A) Representative immunofluorescence staining of caspase-3 active (yellow) and Ki-67 (red) for a whole organoid at day 100

of the culture. Scale bar: 10 µm. **B)** Representative immunofluorescence staining of caspase-3 active (yellow) and Ki-67 (red) for an organoid slice at day 100 of the culture. Scale bar: 20 µm. **C)** Integrated density normalized to the related nuclei area of Ki-67 and caspase-3 active in whole organoids and organoid slices at day 100 of the culture. Data are presented as mean ± SD for three independent experiments (N = 3) and two to three organoids per experiment (n = 2-3), * $p<0.05$, ** $p<0.01$, *** $p<0.001$, **** $p<0.0001$. Statistical analysis was done using unpaired t-test.

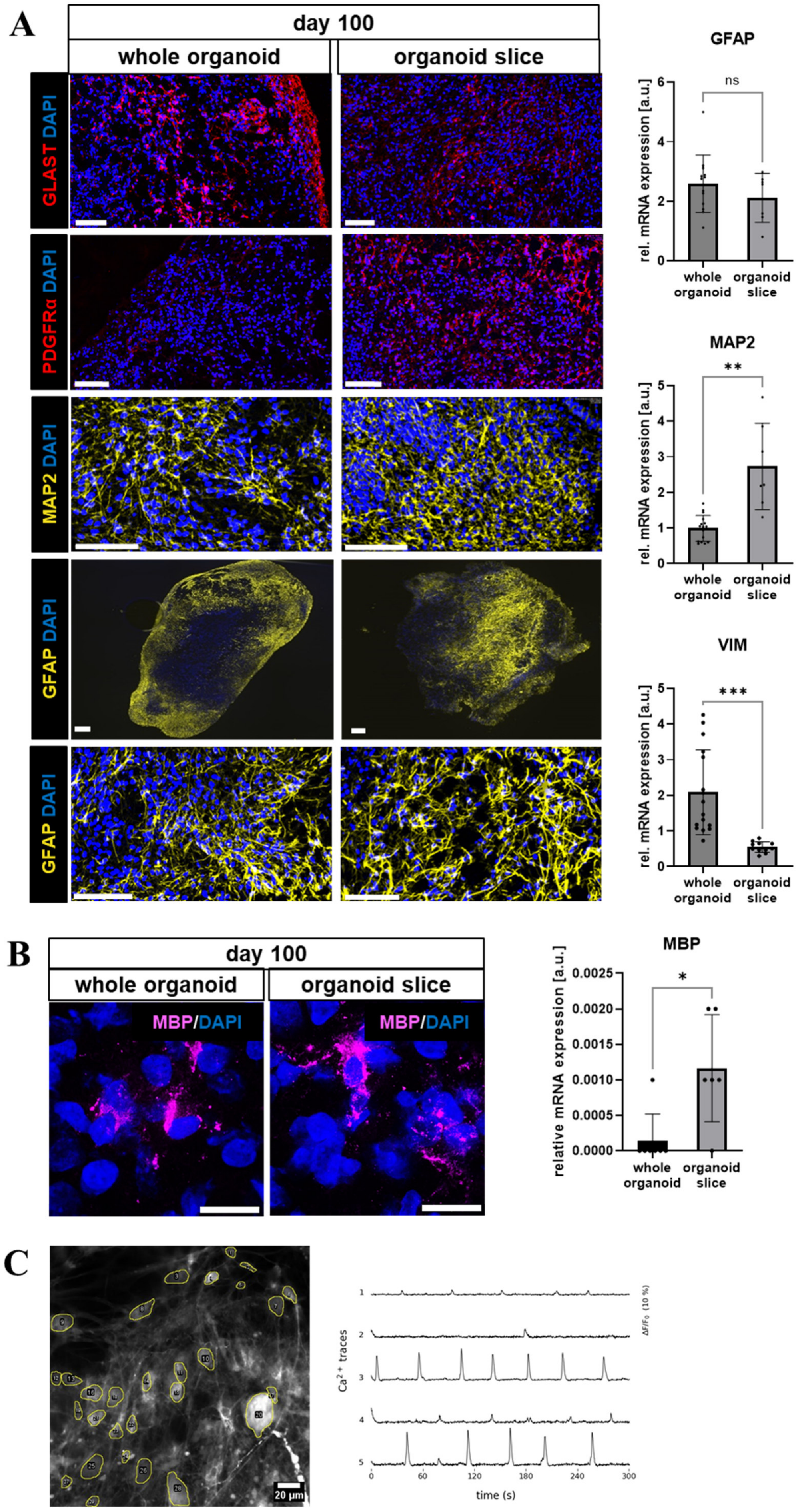
A
day 100
whole organoid
organoid slice
GLAST DAPI
PDGFRα DAPI
MAP2 DAPI
GFAP DAPI
GFAP DAPI
GFAP
ns
rel. mRNA expression [a.u.]
whole organoid
organoid slice
MAP2
**
VIM
***
B
day 100
whole organoid
organoid slice
MBP/DAPI
MBP/DAPI
MBP
*
relative mRNA expression [a.u.]
C
20 µm
Ca2+ traces
ΔF/F0 (10 %)
time (s)

Supplementary Figure 4. Maturation in whole organoids and organoid slices, Related to Figure 3. A) (left side) Representative immunofluorescence staining of GLAST (red), PDGFRα (red), MAP2 (yellow), and GFAP (yellow) for whole organoids and organoid slices at day 100 of the culture. Scale bar: 100 µm, 200 µm for GFAP in whole organoids. (right side) Relative mRNA expression of *GFAP*, *MAP2*, and *VIM* in whole organoids and organoid slices at day 100 of the culture. Data are presented as mean ± SD for three to six independent experiments (N = 3-6) and three organoids per experiment (n = 3) for whole organoids, and N = 6 (or N = 10 for VIM), n = 3 for organoid slices, * p<0.05, ** p<0.01, *** p<0.001, **** p<0.0001. Statistical analysis was done using Welch`s t-test. **B)** (left side) Representative immunofluorescence staining of MBP (magenta) for whole organoids and organoid slices at day 100 of the culture. Scale bar 20 µm. (right side) Relative mRNA expression of MBP in whole organoids and organoid slices at day 100 of the culture. Data are presented as mean ± SD for three to six independent experiments (N = 7) and three organoids per experiment (n = 3) for whole organoids, and N = 6, n = 3 for organoid slices, * p<0.05. Statistical analysis was done using Welch`s t-test. **C)** (left side) Representative image of Ca-imaging of multiple cell bodies (yellow circled) of one organoid slice at day 81 of the culture. Scale bar: 20 µm. (right side) Exemplary Ca-traces of five different cells measured over 300 seconds.

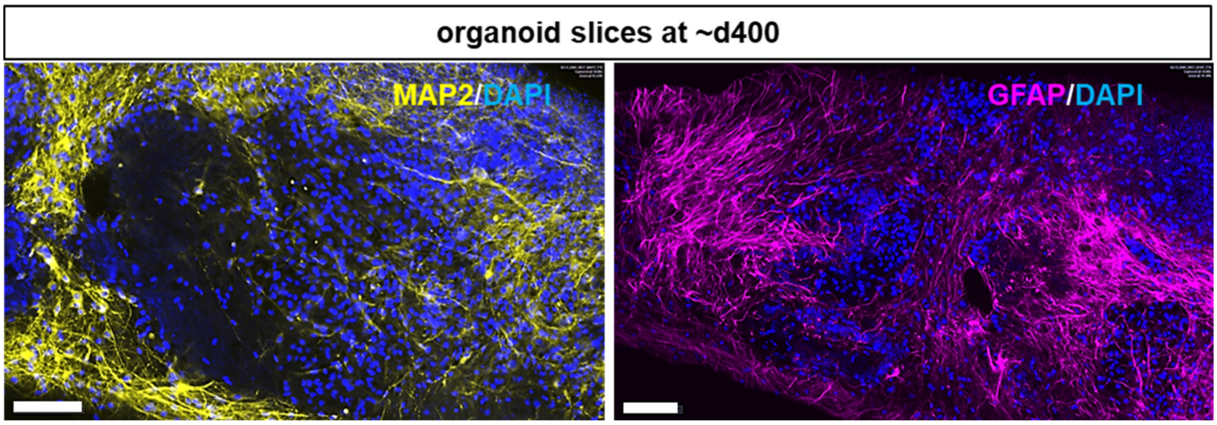

Supplementary Figure 5. **Organoid slices cultured over one year, Related to Figure 4.** Representative immunofluorescence staining of MAP2 (yellow) and GFAP (magenta) for organoid slices at ~day 400 of the culture. Scale bar: 100 µm.

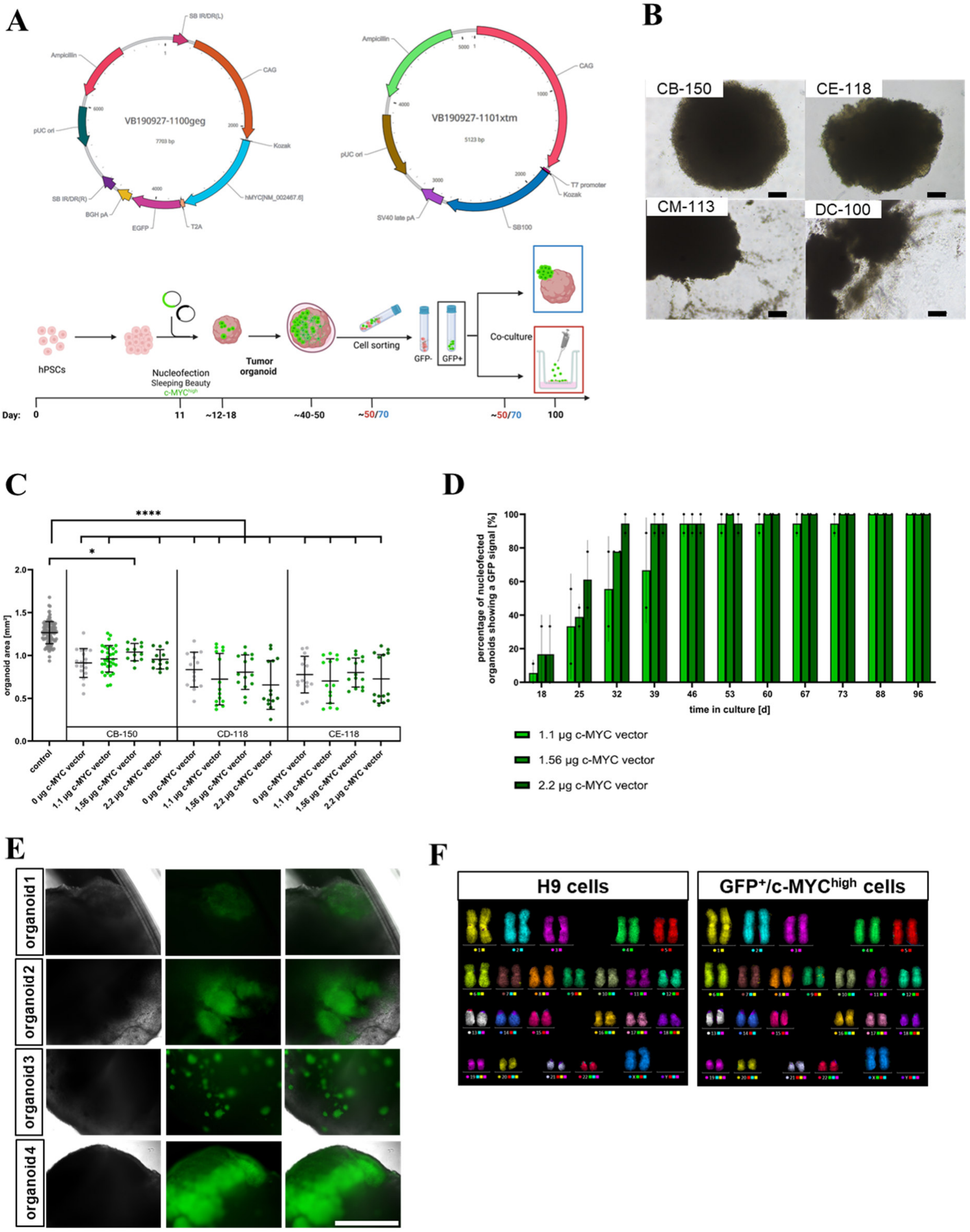

Supplementary Figure 6. Establishment of the genetic modification protocol, Related to Figure 5. A) (top) Vector maps of and *c-MYC* (left, VB190927-1100geg) and *Sleeping Beauty* Transposase *SB100* (right, VB190927-1101xtm). (bottom) Workflow of the genetically modification protocol for the generation of the tumor models. Embryoid bodies were generated using human pluripotent stem cells (hPSCs) and organoids were nucleofected on day 11 of the culture using *Sleeping Beauty* transposon system for overexpression of *c-MYC* oncogene together with *GFP*. GFP$^+$ genetically modified cells were detectable within one week and GFP$^+$ areas increased until isolation of genetically modified cells based on the presence of GFP signal using fluorescence-activated cell sorting at ~day 50 for co-culture with organoid slices or at ~day 70 for generation of tumor spheres to be assembled with whole organoids. Created with BioRender.com. **B)** Organoids 3 days after nucleofection (on day 14 of the

culture) using the program CB-150, CE-118, CM-113, or DC-100 of 4D-Nucleofector™ from Lonza. Scale bar 250 µm. **C)** Organoid size measured by the circular area 7 days after nucleofection (on day 18 of the culture) using the program CB-150, CD-188 or CE-118 of 4D-Nucleofector™ from Lonza. In each case, nucleofection was performed using 0 µg, 1.1 µg, 1.56 µg or 2.2 µg *c-MYC* vector and compared to non-nucleofected control organoids. Data are presented as mean ± SD for two to eight independent experiments (N = 2-8) and 2 to 30 organoids per experiment (n = 2-30), while each point represents the measurement of one single sample. * $p<0.05$, ** $p<0.01$, *** $p<0.001$, **** $p<0.0001$. Statistical analysis was done using Kruskal-Wallis Test with Dunn's post-test. **D)** Percentage of organoids showing a GFP^{+} area after nucleofection with 1.1 µg, 1.56 µg or 2.2 µg *c-MYC* vector using the program CB-150 of 4D-Nucleofector™ from Lonza. Two independent experiments (N = 2) with nine organoids per experiment (n = 9). **E)** Exemplary GFP^{+} areas of 4 different organoids of one experiment on day 53 of the culture. Scale bar: 1000 µm. **F)** Representative normal m-FISH karyotype of H9 cells and GFP^{+}/c-MYC^{high} cells.

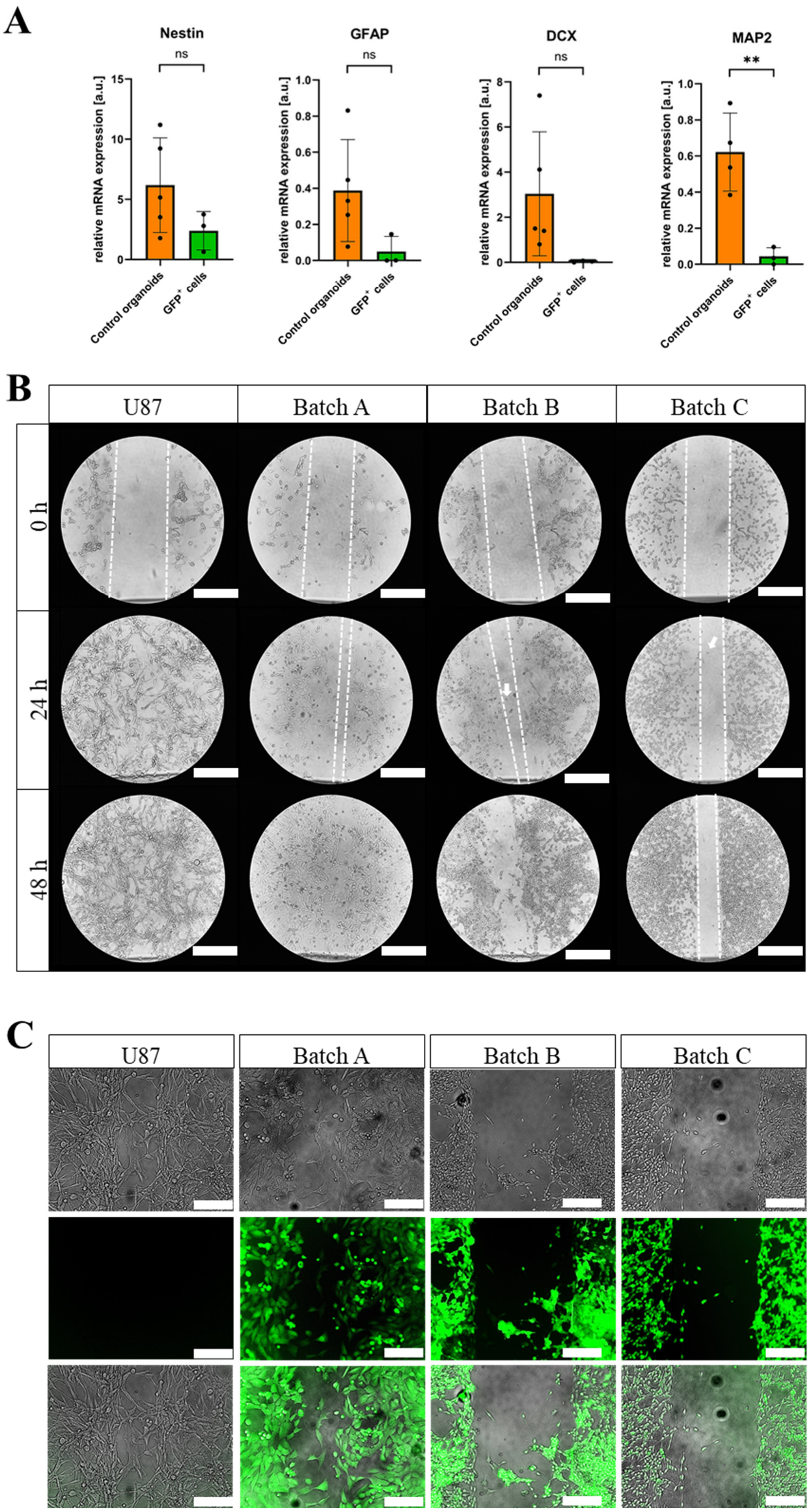
A
Nestin
ns
GFAP
ns
DCX
ns
MAP2
**
relative mRNA expression [a.u.]
Control organoids
GFP⁺ cells
B
U87
Batch A
Batch B
Batch C
0 h
24 h
48 h
C
U87
Batch A
Batch B
Batch C

Supplementary Figure 7. Genetically modified tumor-like cells generated in organoids show immature properties, Related to Figure 5. A) Relative mRNA expression of *NES*, *GFAP*, *MOG*, *DCX*, and *MAP2* in GFP^+ cells compared to whole non-nucleofected control organoids. Data are presented as mean ± SD for two to three independent experiments (N = 2-3) and one to two organoids per experiment (n = 1-2), * $p<0.05$, ** $p<0.01$, *** $p<0.001$, **** $p<0.0001$. Statistical analysis was done using unpaired t-test. **B)** Scratch assay of the glioblastoma cell line U87 and three different batches of isolated GFP^+ cells from three different nucleofection and FACS processes. Representative images of the same positions of the individual scratches were documented 0 h, 24 h, and 48 h after scratching, while the white lines mark the approximated boundaries of the scratches and the white arrows mark single GFP^+ cells that migrated into the scratches. Scale bar: 400 µm. **C)** Representative magnification images of the scratches described in B). Scale bar: 150 µm.

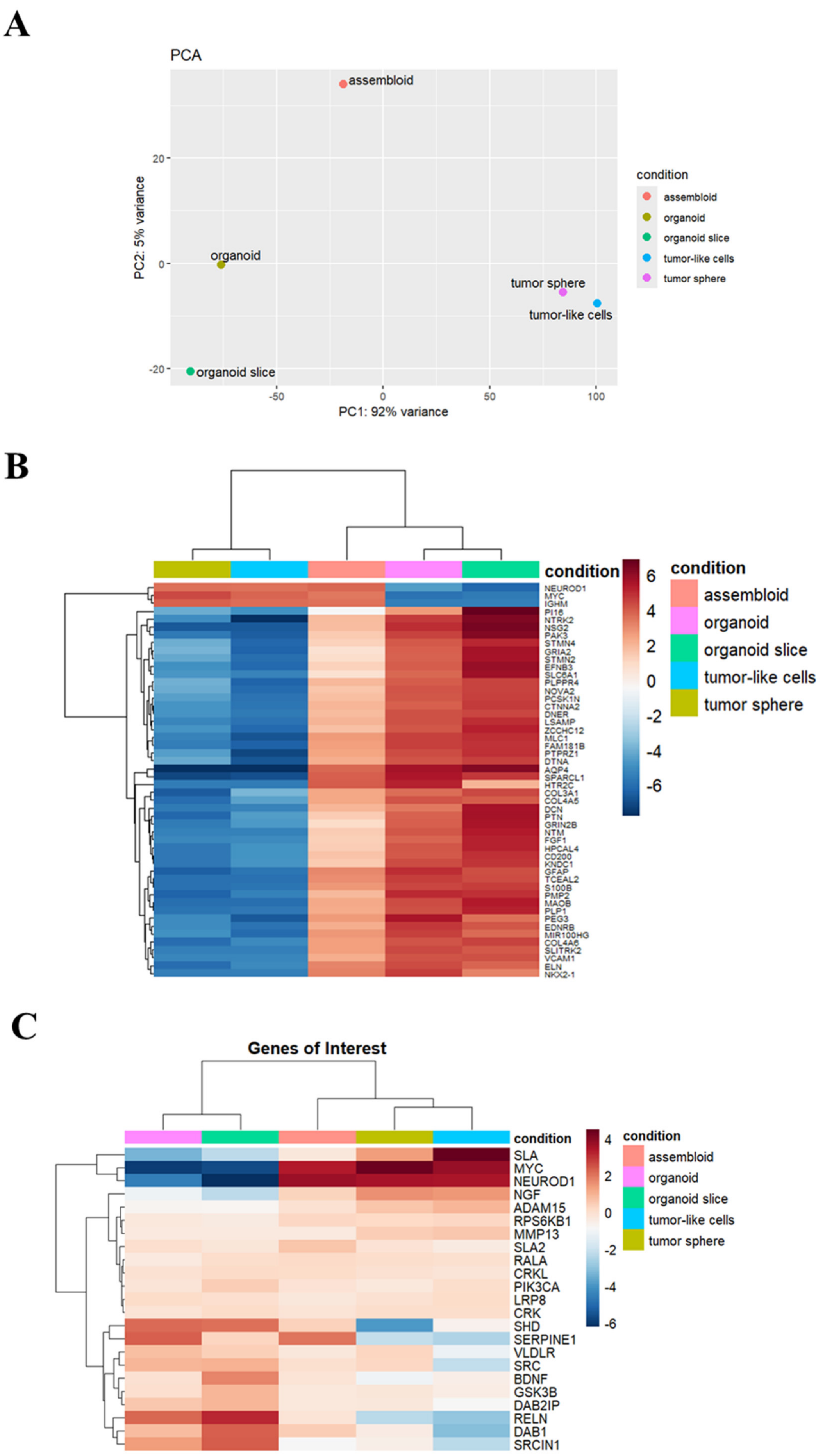
A
PCA
assembloid
organoid
organoid slice
tumor sphere
tumor-like cells
20
0
-20
-50
0
50
100
PC2: 5% variance
PC1: 92% variance
condition
assembloid
organoid
organoid slice
tumor-like cells
tumor sphere
B
condition
NEUROD1
MYC
IGHM
PI16
NTRK2
NSG2
PAK3
STMN4
GRIA2
STMN2
EFNB3
SLC6A1
PLPPR4
NOVA2
PCSK1N
CTNNA2
DNER
LSAMP
ZCCHC12
MLC1
FAM181B
PTPRZ1
DTNA
AQP4
SPARCL1
HTR2C
COL3A1
COL4A5
DCN
PTN
GRIN2B
NTM
FGF1
HPCAL4
CD200
KNDC1
GFAP
TCEAL2
S100B
PMP2
MAOB
PLP1
PEG3
EDNRB
MIR100HG
COL4A6
SLITRK2
VCAM1
ELN
NKX2-1
6
4
2
0
-2
-4
-6
condition
assembloid
organoid
organoid slice
tumor-like cells
tumor sphere
C
Genes of Interest
condition
SLA
MYC
NEUROD1
NGF
ADAM15
RPS6KB1
MMP13
SLA2
RALA
CRKL
PIK3CA
LRP8
CRK
SHD
SERPINE1
VLDLR
SRC
BDNF
GSK3B
DAB2IP
RELN
DAB1
SRCIN1
4
2
0
-2
-4
-6
condition
assembloid
organoid
organoid slice
tumor-like cells
tumor sphere

Supplementary Figure 8. Exploratory bulk RNA sequencing analysis for whole organoids, organoid slices, assembloids, tumor spheres and tumor-like cells (TLC), Related to Figure 8. A) PCA analysis for similarities in gene expression between different culture conditions. **B)** Heat-map showing the top 50 differentially expressed genes. **C)** Heat-map showing the differentially expressed genes of interest. One independent experiment (N = 1) with three to six replicates per group (n = 3-6).

Supplementary Table 1. Primary Antibodies, Related to STAR Methods.

**Antigen**	**Species**	**Company**	**Catalog No.**	**Dilution**	**RRID**
SOX2	rabbit	Thermo Fisher Scientific	A24339	1:100	AB_2924437
Nestin	mouse	BD Biosciences	611658	1:500	AB_399176
DCX	rabbit	Abcam	ab18723	1: 1000	AB_732011
CX43	rabbit	Abcam	ab11370	1:1000	AB_297976
SMI312	mouse	BioLegend	837904	1:300	AB_2566782
GFP	chicken	Thermo Fisher Scientific	A10262	1:300	AB_2534023
VIM	mouse	Thermo Fisher Scientific	14-9897-82	1:100	AB_10597910
CD133	rabbit	Abcam	ab19898	1:200	AB_470302
c-Myc	mouse	Thermo Fisher Scientific	MA1-980	1:100	AB_558470
c-Myc	rabbit	Cell Signaling Technology	13987	1:1000	AB_2631168
Ki-67	rabbit	Abcam	ab16667	1:500	AB_302459
PDGFRα	rabbit	Atlas Antibodies	HPA004947	1:250	AB_2732399
GLAST	rabbit	Thermo Fisher Scientific	PA5-111080	1:250	AB_2856490
MBP	rabbit	Abcam	ab218011	1:500	AB_2895537
SYN1	rabbit	Synaptic Systems	106103	1:1000	AB_11042000
VAMP2	rabbit	Synaptic Systems	104202	1:1000	AB_887810
HOMER1	mouse	Synaptic Systems	160011	1:400	AB_2120992
PSD95	mouse	Synaptic Systems	124 011	1:500	AB_10804286

Supplementary Table 2. Secondary Antibodies, Related to STAR Methods.

Host	Target	Fluor-ophore	Company	Catalog No.	Dilution	RRID
donkey	anti-mouse	AF488	Thermo Fisher Scientific	A24350	1:250	AB_2924437
donkey	anti-rabbit	AF594	Thermo Fisher Scientific	A24343	1:1000	
goat	anti-mouse	AF568	Thermo Fisher Scientific	A11004	1:1000	AB_2534072
goat	anti-rabbit	AF594	Thermo Fisher Scientific	A11012	1:1000	AB_2534079
goat	anti-mouse	AF594	Thermo Fisher Scientific	A21235	1:1000	AB_2535804
donkey	anti-mouse	AF594	Thermo Fisher Scientific	A32744	1:1000	AB_2762826
goat	anti-chicken	AF647	Thermo Fisher Scientific	A32933	1:1000	AB_2762845
goat	anti-mouse	AF647	Thermo Fisher Scientific	A21235	1:1000	AB_2535804
goat	anti-mouse	AF488	Thermo Fisher Scientific	A32723	1:500	AB_2633275

Supplementary Table 3. Directly Labeled Antibodies, Related to STAR Methods.

Antigen	Species	Company	Catalog No.	Dilution	RRID
MAP2	rabbit	Abcam	ab225315	1:300	AB_3517252
GFAP	rabbit	Abcam	ab194325	1:300	AB_3662092
Ki-67	rabbit	Abcam	ab216709	1:300	AB_3662093
Caspase-3 active	rabbit	GeneTex	GTX22302	1:300	AB_384753

Supplementary Table 4. Primers for qRT-PCR, Related to STAR Methods.

Gene	Accession Nr.	Primer sequence (5´- 3´)
18S rRNA	NR_003286.2	ACTCAACACGGGAAACCTCACC (s)
		CGCTCCACCAACTAAGAACGG (as)
c-MYC	NM_002467.6/ NM_001354870.1	TCGGATTCTCTGCTCTCCTC (s)
		CCTGCCTCTTTTCCACAGAA (as)
TP53	NM_001407266.1	CCTCAGCATCTTATCCGAGTGG (s)
		TGGATGGTGGTACAGTCAGAGC (as)
MKI67	NM_002417.5	GTGGTTCGACAAGTGGCCTT (s)
		ACAACTCTTCCACTGGGACG (as)
NF1	NM_000267.3	GGACTCTAAGATCAACACCCTG (s)
		CACCACACTCTGCACAATTCCAT (as)
PTEN	NM_001304718.2	TGAGTTCCCTCAGCCGTTACCT (s)
		GAGGTTTCCTCTGGTCCTGGTA (as)
PROM1	XM_054351160.1	CACTACCAAGGACAAGGCGTTC (s)
		CAACGCCTCTTTGGTCTCCTTG (as)
GLS	XM_054341407.1	CAGAAGGCACAGACATGGTTGG (s)
		GGCAGAAACCACCATTAGCCAG (as)
SNAI1	NM_005985.4	TGCCCTCAAGATGCACATCCGA (s)
		GGGACAGGAGAAGGGCTTCTC (as)
MAP2	NM_002374.3	TGCGCTGATTCTTCAGCTTG (s)
		TGTGTCGTGTTCTCAAAGGGT (as)
GFAP	NM_002055.5	GTACCAGGACCTGCTCAAT (s)
		CAACTATCCTGCTTCTGCTC (as)
VIM	NM_003380.5	AGGCAAAGCAGGAGTCCACTGA (s)
		ATCTGGCGTTCCAGGGACTCAT (as)
MBP	NM_001025081.1	CTGTGCAACATGTACAAGGACTC (s)
		GGGACAGTCCTCTCCCCTTT (as)
DCX	NM_178152.3	AAGGACCTGTACCTGCCTCT (s)
		TGAGCACTCTCCCCTCCTTT (as)
